# Developing a Multifunctional Cathode for Photoassisted Lithium–Sulfur Battery

**DOI:** 10.1002/advs.202402978

**Published:** 2024-07-19

**Authors:** Fei Zhao, Ke Yang, Yuxin Liu, Juan Li, Chan Li, Xinwu Xu, Yibo He

**Affiliations:** ^1^ State Key Laboratory of Solidification Processing Center of Advanced Lubrication and Seal Materials School of Materials Science and Engineering Northwestern Polytechnical University Xi'an Shaanxi 710072 P. R. China

**Keywords:** Au@N‐TiO_2_/CC, electrocatalysis, lithium–sulfur batteries, photocatalysis, photoelectrode

## Abstract

Integration of solar cell and secondary battery cannot only promote solar energy application but also improve the electrochemical performance of battery. Lithium–sulfur battery (LSB) is an ideal candidate for photoassisted batteries owing to its high theoretical capacity. Unfortunately, the researches related the combination of solar energy and LSB are relatively lacking. Herein, a freestanding photoelectrode is developed for photoassisted lithium–sulfur battery (PALSB) by constructing a heterogeneous structured Au@N‐TiO_2_ on carbon cloths (Au@N‐TiO_2_/CC), which combines multiple advantages. The Au@N‐TiO_2_/CC photoelectrode can produce the photoelectrons to facilitate sulfur reduction during discharge process, while generating holes to accelerate sulfur evolution during charge process, improving the kinetics of electrochemical reactions. Meanwhile, Au@N–TiO_2_/CC can work as an electrocatalyst to promote the conversion of intermediate polysulfides during charge/discharge process, mitigating induced side reactions. Benefiting from the synergistic effect of electrocatalysis and photocatalysis, PALSB assembled with an Au@N‐TiO_2_/CC photoelectrode obtains ultrahigh specific capacity, excellent rate performance, and outstanding cycling performance. What is more, the Au@N‐TiO_2_/CC assembled PALSB can be directly charged under light illumination. This work not only expands the application of solar energy but also provides a new insight to develop advanced LSBs.

## Graphical abstract

Photoassisted lithium–sulfur battery (PALSB) can promote the solar energy application. A freestanding Au@N‐TiO_2_/carbon cloths (CC) photoelectrode for PALSB cannot only work as a photocatalyst that produces the electrons and holes to facilitate the reduction and evolution of sulfur, but also can work as an electrocatalyst to promote the conversion of intermediate polysulfides during charge/discharge process.

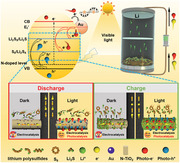

## 1 Introduction

The massive burning of fossil fuels leads to excessive greenhouse gas emissions, resulting in serious environmental pollution.^[^ ^1^ ^]^ Developing environmentally friendly sustainable energy supply and storage systems is of great significance for achieving carbon neutral.^[^ ^2^ ^]^ Solar energy, the source of almost all energy on earth has aroused human interest in research because of its low cost, environmental friendliness and abundance.^[^ ^3^ ^]^ However, due to the changing solar cycle and fluctuating sunlight intensity, solar energy output is discontinuous both temporally and spatially, making it impossible to be a stable energy supply.^[^ ^4^ ^]^ Converting solar energy into electrical energy and storing it is an effective way to mitigate the influence of solar energy fluctuations.^[^ ^5^ ^]^ An energy storage device integrating solar cell and secondary battery has been aroused widespread interest as it can simultaneously realize the conversion and storage of solar energy. Specifically, light energy is converted into electrical energy through solar energy, and the electrical energy is converted into chemical energy during the charging process of secondary batteries, then releases it as electric energy when needed.^[^ ^6^ ^]^ Unfortunately, complex conversion process will cause the solar energy to suffer serious energy loss.

Nowadays, photo rechargeable batteries have been proposed as a promising approach for solar energy application.^[^ ^7^ ^]^ For example, Narayanan's group successfully constructed photorechargeable li‐Ion batteries by employing a TiS_2_–TiO_2_^[^ ^7a^ ^]^ composite and a nanorod heterostructure MoS_2_/MoO*_x_* ^[^ ^7b^ ^]^ as electrode. In addition, Michael de Volder's group developed photorechargeable zinc‐ion batteries by using MoS_2_/ZnO^[^ ^7c^ ^]^ and V_2_O_5_^[^ ^7d^ ^]^ photoactive cathode materials. Inspired by these works, the photoassisted rechargeable batteries have also been developed as a hopeful strategy to introduce solar energy into rechargeable batteries. Among various rechargeable batteries, lithium–sulfur batteries (LSBs) stand out owing to the ultrahigh theoretical energy density (2600 Wh kg^−1^), high safety and low cost.^[^ ^8^ ^]^ However, the electrochemical performance of conventional LSBs is impacted by low utilization and slow conversion of active materials. What's worse, the shuttle effect of long‐chain polysulfides generated during charging and discharging also leads to poor rate performance and cycle stability of LSBs, thus hindering the practical application of LSBs.^[^ ^9^ ^]^ Introducing polar materials to confine sulfur in the cathode is considered as one of the most effective strategies to trap the polysulfides via physical adsorption/chemical bonding. Moreover, the polar materials also can be used as electrocatalysts to promote the conversion of polysulfides.^[^ ^10^ ^]^ Nevertheless, because of the limited active sites and internal space, the structure of cathode materials would be gradually cracked with the polysulfides accumulation, especially in the case of high sulfur loading.^[^ ^11^ ^]^ What's worse, the insulating nature of most polar materials requires high content of conductive additives, which inevitably sacrifice the overall energy density of battery.^[^ ^12^ ^]^ Fortunately, a photoelectrode can produce photoelectric effect, photocatalytic effect and photoconductive effect by the excitation of solar light, which are beneficial to the charge transfer and promote the electrochemical reactions of the rechargeable batteries.^[^ ^13^ ^]^ It means that a rational designed photoelectrode can promote the electrochemical reaction kinetics of LSBs through the synergistic effect of photocatalysis and electrocatalysis. Zhou group first fabricated a Pt/CdS photocatalyst with an aqueous polysulfide cathode to realize the conversion and storage of solar energy in the LSBs, obtaining about five times the specific capacity of conventional lithium‐ion batteries.^[^ ^14^ ^]^ Chen et al. assembled three perovskite solar cells for LSBs charging and achieved an overall energy conversion efficiency of 5.14% with good stability.^[^ ^15^ ^]^ Liu et al. constructed PALSB by using a CdS–TiO_2_ cathode, which decreased the reaction energy barrier of polysulfides to Li_2_S by 70 mV via the photocatalysis effect derived from the photogenerated electrons/holes. Meanwhile, the photoconductive effect largely increases the charge concentration and boosts the electrochemical kinetics because of abundant photogenerated carriers, achieving a high‐performance PALSB.^[^ ^16^ ^]^ Li et al. proved that the electrons in a hybrid S/N719 dye cathode can be captured by the holes of the dye molecule, which accelerates the oxidation of polysulfide, speeds up the charge rate of the cathode, and significantly reduces the charging time.^[^ ^17^ ^]^ In addition, an all solid‐state photorechargeable LSB system for indoor energy harvesting and storage was successfully constructed by using an all‐inorganic CsPbI_2_Br perovskite solar cell module.^[^ ^18^ ^]^ This work promoted the application of photocatalysis in LSBs, and greatly improved the performance of LSBs. Unfortunately, there are still few studies on PALSB, and the relevant mechanism is not yet clear. More photoelectrodes that can be employed in PALSB still need to be excavated and studied.

Herein, we developed a freestanding photoelectrode by constructing a heterogeneous structured Au@N‐TiO_2_ on carbon cloths for PALSB. Attributed to the exceptional photon capture ability and electron–hole separation rate of Au@N‐TiO_2_/CC photoelectrode, it can produce the photoelectrons to facilitate the sulfur reduction reaction during the discharge process while the generated holes accelerate the sulfur evolution reaction during the charge process, realizing a photocatalytic effect. The Au@N‐TiO_2_/CC assembled PALSB integrates photocatalysis and electrocatalysis, which significantly enhances the utilization of active materials to obtain an ultrahigh specific capacity of 1667 mA h g^−1^. Meanwhile, the generated carriers induced by the Au@N‐TiO_2_/CC under illumination can increase the free electron concentration of the battery system, thus decreasing the resistance and improving the electrochemical dynamic behavior, achieving an excellent rate performance. Moreover, the PALSB with Au@N‐TiO_2_/CC photoelectrode can be directly charged under light illumination without applying external circuit voltage, obtaining a discharge specific capacity of 179 mA h g^−1^, which reaches the level of conventional lithium‐ion batteries.

## 2 Results and Discussion

### 2.1 Design Principal of Au@N‐TiO_2_/CC Photoelectrode

**Figure 1:**
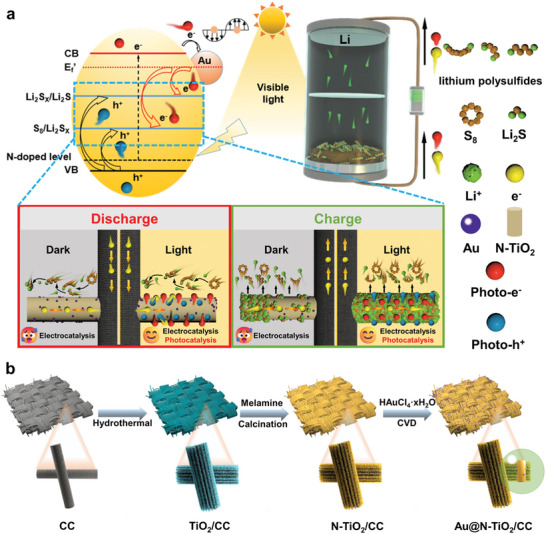
a) Catalytic mechanism of an Au@N‐TiO_2_/CC photoelectrode in a PALSB. b) Schematic illustrating the fabrication progress of Au@N‐TiO_2_/CC photoelectrode.

A schematic diagram illustrates the design thought and working mechanism of a PALSB with Au@N‐TiO_2_/CC electrode. As shown in **Figure** **1**a, the photogenerated electrons are excited under visible light irradiation, leaving holes in the valance band (VB) and generating electron–hole pairs. This promotes the redox reaction of sulfur during the charge and discharge process. N‐doping narrows the band gap of TiO_2_ by the formation of N 2p states above the VB, extending the light response to the visible region and promoting the efficiency of charge transfer.^[^ ^19^ ^]^ However, N doping has some limitations with limited activity enhancement in visible light, which can be improved by codoping. Noble metals usually have strong catalytic effect, and Au nanoparticles show great potential in the field of photocatalysis. On the one hand, the local surface plasmon resonance effect of noble metal widens the light response range of composite structures when combining with TiO_2_. Au nanoparticles are excited under visible light and generate hot electrons.^[^ ^20^ ^]^ On the other hand, TiO_2_ is an n‐type semiconductor whose work function is smaller than that of Au. When they contact, the electrons of TiO_2_ flow to Au nanoparticles until their Fermi levels are equal, and the energy bands bend, forming Schottky junctions that accelerate carrier separation and inhibit recombination.^[^ ^21^ ^]^ Due to the existence of Schottky barrier, photogenerated electrons are relatively enriched on Au and react with oxidants for reduction, while the holes left in TiO_2_ are oxidized. This approach prevents the reduction and the oxidation reactions from occurring on the same grain surface, ensuring that the two reactions are spatially separated. Au nanoparticles also act as electron traps, reducing the probability of photogenerated electron–hole recombination, and promoting carrier transfer.^[^ ^22^ ^]^

Figure S1 (Supporting Information) illustrates the assembly strategy of PALSB. Specifically, sunlight is collected through a transparent window sealed with epoxy resin glue to ensure the stability of the internal system of LSBs. The conversion mechanism of TiO_2_/CC and Au@N‐TiO_2_/CC electrodes is shown in Figure S2a,b (Supporting Information). Polar material TiO_2_ has strong polysulfides adsorption capacity, but its poor conductivity results in slow redox kinetics. In addition, photoassisted effect for polysulfides is limited by the low visible light activity due to the wide band gap of TiO_2_.^[^ ^23^ ^]^ As mentioned previously, the defects of TiO_2_ are alleviated through Au and N codoping. The Au@N‐TiO_2_/CC electrode exhibits a strong adsorption and catalytic conversion abilities for polysulfides and generates a significant number of carriers when illuminated, providing more electron transfer channels. This accelerates the sulfur reduction reaction during the discharge process and enables more Li_2_S to be deposited along the nanorods. The preparation process of a freestanding Au@N‐TiO_2_/CC photoelectrode is shown as Figure 1b. Specifically, uniform and dense TiO_2_ nanorods are firstly grown on carbon cloth (CC) by hydrothermal method. Then the N‐TiO_2_/CC electrode is obtained through a melamine coordination heat treatment process. Finally, Au@N‐TiO_2_/CC multifunctional photoelectrode is produced by introducing HAuCl_4_⋅*x*H_2_O as the source for Au nanoparticles via a chemical vapor deposition (CVD) process.

### 2.2 Structure and Morphology of Au@N‐TiO_2_/CC

**Figure 2:**
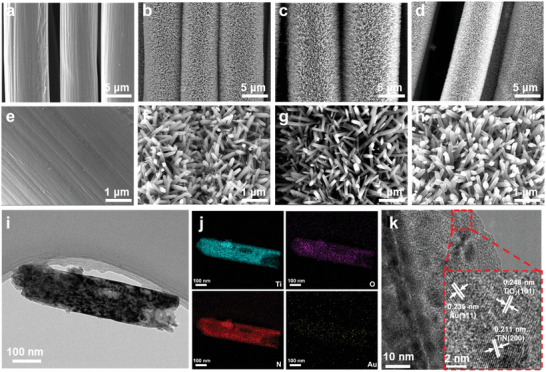
SEM images of a,e) CC; b,f) TiO_2_/CC; c,g) N‐TiO_2_/CC; d,h) Au@N‐TiO_2_/CC. i) TEM; j) Elemental mapping; k) HRTEM of Au@N‐TiO_2_/CC.

The morphology evolution of the electrode is characterized by SEM. It can be seen that the CC is woven from smooth carbon fiber with a diameter of about 10 µm (**Figure** **2**a,e and F igure S3, Supporting Information). After the hydrothermal treatment, the smooth surface of CC is uniformly covered with TiO_2_ nanorods (Figure 2b,f), which is further demonstrated by the elemental mapping of TiO_2_/CC shown in Figure S4 (Supporting Information). N‐doped TiO_2_ still remains nanorods structure (Figure 2c,g), and the elemental mapping confirms the successful doping of N element (Figure S5, Supporting Information). The morphology of Au decorated N‐TiO_2_ nanorods is shown in Figure 2d,h. The ordered structure provides sufficient capacity to store the reaction products and buffer the volume expansion that occurs during the reaction. TEM (Figure 2i) and HAADF (Figure S6, Supporting Information) show the successful attachment of Au nanoparticles to N‐TiO_2_ nanorods, resulting from the Schottky junction formed between TiO_2_ and Au nanoparticles.^[^ ^24^ ^]^ The element diagram (Figure 2j and Figure S7, Supporting Information) shows that Ti, O, N and Au elements are uniformly distributed on the surface of Au@N‐TiO_2_/CC, confirming the successful construction of a heterostructure Au@N‐TiO_2_/CC. The lattice spacing in HRTEM images is 0.211, 0.235, and 0.248 nm, corresponding to the (200) plane of TiN, (111) plane of Au and (111) plane of TiO_2_, respectively, which further verifies the effective growth of Au@N‐TiO_2_/CC heterostructure (Figure 2k).

**Figure 3:**
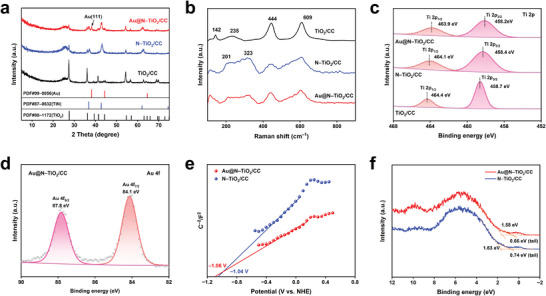
a) XRD patterns, b) Raman curves, and c) Ti 2p XPS spectra of TiO_2_/CC, N‐TiO_2_/CC and Au@N‐TiO_2_/CC. d) Au 4f XPS spectrum of Au@N‐TiO_2_/CC. e) Mott‐Schottky plots and f) XPS valence band spectra of N‐TiO_2_/CC and Au@N‐TiO_2_/CC.

The structure of the different materials is revealed by the X‐ray diffraction (XRD), Raman and X‐ray photoelectron spectrum (XPS) (**Figure** **3**). It can be seen that the XRD pattern of TiO_2_/CC shows a pure rutile phase, and the peaks of N‐TiO_2_/CC at 36.8°, 42.8°, 62.1° match well with the TiN. The nitrogen replaced some oxygen sites of TiO_2_ and the N doped TiO_2_ synthesized at high temperatures had a good crystallinity, hence TiN characteristic peaks appeared on the corresponding XRD patterns. And a new peak appearing at 38.2° of Au@N‐TiO_2_/CC corresponds to the (111) plane of Au (Figure 3a). For the Raman spectra, the characteristic vibration peaks of N‐TiO_2_/CC at 201 and 323 cm^−1^ also prove the successful doping of N (Figure 3b). Au nanoparticles adhere to the surface of TiO_2_, forming Schottky junction. According to Schottky function, some oxygen vacancies occur at the metal–semiconductor interface.^[^ ^25^ ^]^ Compared with N‐TiO_2_/CC, peak shift is observed in the Raman spectra of Au@N‐TiO_2_/CC (Figure S8, Supporting Information). The full XPS spectra of the materials are shown in Figure S9 (Supporting Information). It can be seen that the Ti 2p_1/2_ and Ti 2p_3/2_ in N‐TiO_2_/CC and Au@N‐TiO_2_/CC shifted to 464.1, 458.4, and 463.9, 458.2 eV respectively, and N 1s in Au@N‐TiO_2_/CC shifted slightly to 396.8 and 400.0 eV respectively, indicating the successful construction of Au@N‐TiO_2_/CC heterostructure (Figure 3c and Figure S10, Supporting Information). The Au 4f_5/2_ and Au 4f_7/2_ peaks also confirmed the attachment of Au (Figure 3d).

The complete electron band structure of the Au@N‐TiO_2_/CC is measured by XPS valence band (XPS‐VB) spectra and Mott‐Schottky (M–S) plot (Figure S11, Supporting Information). To illustrate the properties of the heterojunction, the M–S plot is recorded. The positive slope of the M–S plot suggests the n‐type semiconducting nature of the TiO_2_/CC, N‐TiO_2_/CC and Au@N‐TiO_2_/CC with electrons as the majority carrier (Figure 3e and Figure S12a, Supporting Information). The flat‐band potential (*E*_FB_) of Au@N‐TiO_2_/CC is estimated to be ‐1.06 V, indicating that the conduction band (CB) potential is about ‐1.16 V (vs NHE). Figure 3f and Figure S12b (Supporting Information) show the VB maximum (VBM) edge of TiO_2_/CC to be located at about 2.77 eV. For N‐TiO_2_/CC and Au@N‐TiO_2_/CC, the main absorption onsets are located at about 1.63 and 1.58 eV. Due to the band bending caused by the interfacial charge transfer, the VBM at the band tail are blue‐shifted toward to 0.74 eV and 0.66 eV, respectively.^[^ ^26^ ^]^ The Photoluminescence (PL) emission spectra of semiconductors are shown in Figure S13 (Supporting Information). It can be observed that the emission intensity of N‐TiO_2_/CC and Au@N‐TiO_2_/CC is much lower than TiO_2_/CC, indicating that the introduction of Au and N elements reduces the recombination rate of carriers. Meanwhile, the carrier concentration of Au@N‐TiO_2_/CC and N‐TiO_2_/CC is estimated to be 1.36 × 10^23^ and 0.89 × 10^23^ cm^‐3^, respectively, which confirms the enhanced charge carrier transport and improved photocatalytic activity when Au was decorated on the surface of N‐TiO_2_. Specifically, photogenerated electrons are captured by oxygen vacancies and Au, and photogenerated holes are captured by doped nitrogen.^[^ ^27^ ^]^ In addition, the Schottky barrier between Au and semiconductor is conducive to the transfer of photogenerated electrons and inhibits their recombination.

### 2.3 Electrochemical Kinetic Analysis of Au@N‐TiO_2_/CC Photoelectrode

**Figure 4:**
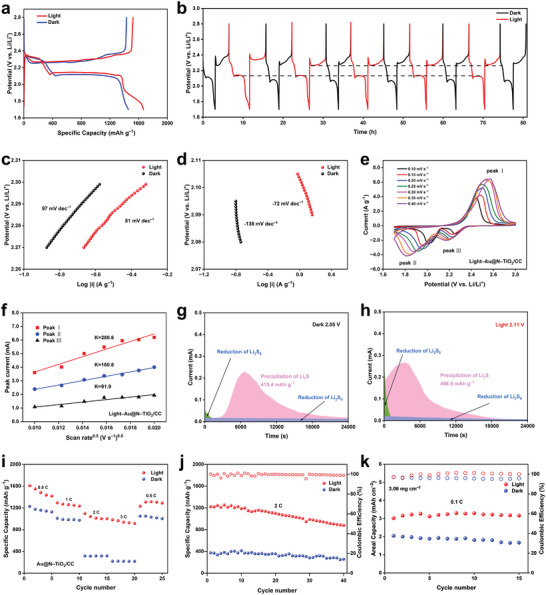
Electrochemical performance of Au@N‐TiO_2_/CC photoelectrode with or without the illumination: a) GCD curves at 0.1 C; b) GCD curves with periodic control of light source switch; c,d) Tafel curves; e) CV curves at different sweep rates; f) Plots of CV peak current versus the square root of the scan rate with illumination. Potentiostatic discharge curves of Li_2_S_8_ catholyte g) without, and h) with the illumination. i) Rate performance; j) cycling performance at 2 C; k) cycling performance at 0.1 C with a high sulfur loading of 3.06 mg cm^‐2^.

To evaluate the adsorption ability of Au@N‐TiO_2_ to polysulfides, Li_2_S_6_ adsorption experiments were conducted. Approximate 20 mg of different samples were added into the 2 mmol L^−1^ Li_2_S_6_ solution and then the change of solution color was recorded. As shown in Figure S14 (Supporting Information), it can be seen that the yellow color of the solution with Au@N‐TiO_2_/CC gradually became colorless with the increase of standing time. On the contrary, the color of the solution with pure CC had no significant change, nor in blank Li_2_S_6_ solution. Moreover, the same results are obtained in Ultraviolet–visible (UV–vis) spectra. Specifically, the intensity of Li_2_S_6_ peak of the solution containing Au@N‐TiO_2_/CC decreased obviously, confirming the excellent adsorption ability of Au@N‐TiO_2_/CC on Li_2_S_6_. To investigate the effect of the photo on the electrochemical performance of PALSB, the Au@N‐TiO_2_/CC electrode is assembled into LSBs with Li metal anode. The active material is dripped on the surface of cathode in the form of 0.5 m Li_2_S_6_ solution. **Figure** **4**a shows the galvanostatic charge/discharge (GCD) curve of the Au@N‐TiO_2_/CC battery at 0.1 C (1 C = 1675 mA g^−1^) for the first cycle with and without the light illumination. A high reversible capacity of 1667 mA h g^−1^ is achieved under illumination. The specific capacity is almost close to the theoretical value and is much higher than that without illumination (1457 mA h g^−1^), indicating that photogenerated electron–hole pairs promote the redox reaction of sulfur during the charge/discharge process. Furthermore, the contribution capacity of pure Au@N‐TiO_2_ cathode without Li_2_S_6_ to the PALSB is investigated. The electrochemical performance of the battery assembled by an Au@N‐TiO_2_ cathode without Li_2_S_6_ is shown in Figure S15a–c (Supporting Information). The specific discharge capacity of Au@N‐TiO_2_/CC electrode under light is less than 16 mA h g^−1^ and then decays to about 10 mA h g^−1^ in 40 cycles, implying that the capacity contribution of Au@N‐TiO_2_/CC cathode to the PALSB is almost negligible. During the discharge process, the photogenerated electrons excited in CB are able to reduce S_8_ to Li_2_S and the holes left in VB are reduced by electrons from the external circuit. During the charge process, the photogenerated holes in VB promote the oxidation of Li_2_S to S_8_, and the electrons reach to the anode through the external path, thus reducing Li^+^ to Li.^[^ ^16^ ^]^ Therefore, the polarization voltage of charge–discharge curve decreased by 6 mV. In addition, the polarization voltages at 0.5 and 1 C decreased by 60 and 158 mV, respectively (Figure S16a–c, Supporting Information). Meanwhile, the charge and discharge plateau are prolonged under light irradiation. These phenomena can be attributed to photocatalytic effect. The key of this process is that the reaction potential of the whole redox reaction of LSBs locate between the CB and VB of semiconductor.^[^ ^28^ ^]^ It is worth noting that the discharge platform has a relatively obvious improvement, which is related to the band structure of the semiconductor. The CB of Au@N‐TiO_2_/CC is about 1.88 V (vs Li/Li^+^), and the VB potential is calculated to be 4.62 V. The reaction potential of the sulfur redox reaction is closer to the CB of the catalyst than the VB. Therefore, photogenerated electrons are more likely to participate in the sulfur redox reaction than photogenerated holes. The potential of the sulfur redox reaction is adequately covered by the band energy of Au@N‐TiO_2_/CC photoelectrode, indicating that the photogenerated electrons/holes possess sufficient redox ability to accelerate the conversion of polysulfides effectively, thereby improving the electrochemical performance of PALSB (Figure S17, Supporting Information). The morphology of the cathode after the first cycle of discharge was observed (Figure S18a,b, Supporting Information). Without illumination, the discharge products accumulated on the surface of the nanorods to form large aggregates, impending the contact between the electrolyte and the active sites, and hindering the further electrochemical reactions. After introducing the illumination, the discharge products deposited along the nanorods and distributed evenly to form a flat surface upon the Au@N‐TiO_2_/CC photoelectrode. It is noteworthy that some void spaces can be observed between the discharge particles, which is beneficial for facilitating the ion transport and will lead to a substantially enhanced electrochemical kinetics of the subsequent charge process. In order to study the effect of light‐assisted charging mechanism on the energetics of LSBs, more related experiments were analyzed. As shown in Figure S19a (Supporting Information), the charging voltage drops sharply when the battery is exposed to light during charging, indicating that the holes generated by Au@N‐TiO_2_/CC photoelectrode can reduce the energy barrier of the sulfur evolution reaction in the presence of light. In addition, the photoassisted charging mechanism of Au@N‐TiO_2_/CC battery is further investigated by XPS analysis of Au@N‐TiO_2_/CC electrodes in fully charged state with and without the illumination. As depicted in Figure S19b,c (Supporting Information), it can be seen that the peak area of the lithium polysulfides decreased from 7.5% to 6.8%, and the characteristic peak area of S_8_ increased from 1.6% to 3.1% after introducing the light illumination, indicating that photogenerated holes efficiently promoted the conversion of polysulfides to S_8_. Meanwhile, the contend of Au increased after charging as more polysulfides on the surface are oxidized with the light illumination. These research results suggest that the photogenerated holes can accelerate the conversion of Li_2_S to S_8_, exhibiting a photocatalytic effect.

Figure 4b shows the charge and discharge potential versus time curves with and without alternating illumination. The discharge voltage platform under light illumination is 2.13 V. After removing the light source, the discharge voltage platform drops to 2.07 V, and the discharge capacity decreases. At the same time, the average charge voltage is increased from 2.29 to 2.32 V, indicating that the Au@N‐TiO_2_/CC photoelectrode reduces the energy barrier of the sulfur redox reaction in the presence of light. After about 80 h photoperiod GCD tests, the change of discharge potential caused by illumination still exists, which shows that Au@N‐TiO_2_/CC cathode can steadily improve the electrochemical kinetics of LSBs. The electrochemical impedance spectra (EIS) of Au@N‐TiO_2_/CC battery are shown in Figure S20a (Supporting Information). There are two semicircles in the high and mid‐frequency regions and the straight line in the low‐frequency region of the Nyquist plot. In the equivalent circuits, *R*_1_ is the impedance of electrolyte resistance; *R*_2_ corresponds to the deposition of lithium polysulfide on the surface of the sulfur cathode, *R*_3_ stands for the charge transfer resistance and W_1_ refers to the Warburg impedance.^[^ ^29^ ^]^ The *R*_1_, *R*_2,_ and *R*_3_ of the battery under light illumination are 7.2, 13.1, and 3.4 Ω, respectively, which are smaller than that without the light illumination (9.9, 88.1, and 7.1 Ω, respectively), suggesting a significant improvement in electron transport and electrochemical kinetics and demonstrating the positive effect of light (resistance fitting parameters are shown in Table S1, Supporting Information). The cyclic voltammetry (CV) curves of Au@N‐TiO_2_/CC battery were tested at a scan rate of 0.1 mV s^−1^ with the voltage window of 1.7–2.8 V (Figure S20b, Supporting Information). The two reduction peaks at CV curves correspond to the reduction of S_8_ to polysulfides intermediates and further conversion to Li_2_S while the oxidation peak relates to the reverse reaction of Li_2_S to S_8_. When illuminated, the reduction peak exhibited higher initial and peak voltages, while the oxidation peak shows lower initial and peak voltages. Meanwhile, the corresponding current value increases, indicating improved kinetic performance during the liquid–solid conversion reaction stage. The initial two cycles of CV curves at 0.1 mV s^−1^ of the Au@N‐TiO_2_/CC assembled battery are shown in Figure S21a,b (Supporting Information). The peak reduction of the first cycle is significantly greater than that of the second cycle, which is attributed to the contribution of the solid electrolyte interface (SEI) layer, indicating that the SEI can be formed with or without the light illumination.^[^ ^30^ ^]^ To analyze the catalytic effect during the sulfur redox reaction of the Au@N‐TiO_2_/CC cathode, the Tafel curves are shown in Figure 4c,d. The Tafel slope is 97 mV dec^−1^ (‐138 mV dec^−1^) without illumination and 81 mV dec^−1^ (‐72 mV dec^−1^) with illumination. The lower the Tafel slope, the better the catalytic activity.^[^ ^31^ ^]^ Thus, Au@N‐TiO_2_/CC exhibits high catalytic activity in accelerating the conversion of polysulfides under illumination.

In order to further investigate the effect of light on electrochemical kinetic of LSBs, the Li^+^ diffusion coefficient was calculated from Equation S1 (Supporting Information) according to the corresponding CV curves at different sweep rates from 0.1 to 0.4 mV s^−1^ (Figure 4e and Figure S22a, Supporting Information). It is obvious that the curve slope of Au@N‐TiO_2_/CC battery with the illumination is much larger than that without the illumination, indicating that photogenerated carriers improve the diffusion ability of Li^+^ (Figure 4f and Figure S22b, Supporting Information). Hence, it can be concluded that the photoconductivity effect of photogenerated electrons and holes increases the carrier concentration of Au@N‐TiO_2_/CC electrode, reducing the resistance and improving the kinetics of the system, thus improving the electrochemical performance of LSBs. To further investigate the contribution of photocatalysis to the redox kinetics of polysulfides, Li_2_S precipitation experiments were designed on the surface of Au@N‐TiO_2_/CC using 0.25 m Li_2_S_8_ cathode electrolyte as active substance. The battery without illumination was first discharged at a constant current of 112 µA to 2.06 V, and then discharged at a constant voltage of 2.05 V for 24 000 s, while the PALSB could be precipitated at 2.11 V, and the precipitation capacity of Li_2_S increased from 415.4 to 498.5 mA h g^−1^. Meanwhile, the redox kinetics is highly accelerated with the earliest nucleation time of 3080 s under illumination (6122 s under dark) (Figure 4g,h). These results indicate that Au@N‐TiO_2_/CC possesses excellent catalytic activity for promoting Li_2_S deposition under illumination. The deposition pattern of Li_2_S is revealed by a dimensionless current–time transient (Figure S23, Supporting Information). In the absence of light illumination, the nucleation of Li_2_S is corresponding to the 2D progressive (2DP) while the Li_2_S deposition with the light illumination is denoted as 2D instantaneous (2DI) model. It clearly shows that the nucleation sites of Li_2_S and the nucleation efficiency are increased after introducing the light into the system.^[^ ^32^ ^]^ Photogenerated electrons increase the conductivity and surface charge concentration of cathode, facilitating the transformation of polysulfide to Li_2_S and the deposition of Li_2_S. Figure 4i illustrates the rate performance of the Au@N‐TiO_2_/CC battery from 0.5 to 3 C with and without the illumination. As the current rate increases, the capacity without illumination rapidly decays, only providing a specific capacity of 217 mA h g^−1^ at 3 C. Fortunately, PALSB can provide a specific capacity of 982 mA h g^−1^ at 3 C, about 4.5 times of that without illumination. When the current drops abruptly from 3 to 0.5 C, a satisfactory capacity of 1232 mA h g^−1^ is still available. It is confirmed that Au@N‐TiO_2_/CC battery has excellent rate performance under light illumination, demonstrating the positive effect of photoassisted effect on LSBs. The charge storage mechanism is analyzed by studying the kinetics of the Au@N‐TiO_2_/CC electrode according to fit the relation between the current (*i*) and scan rate (*v*): *i* = *av^b^*. Specifically, there are three types of charge storage mechanisms: the diffusion controlled faradaic contribution, pseudocapacitive effect of surface charge transfer, and non‐faraday process controlled by electrical double layers.^[^ ^33^ ^]^ The value of *b* can be obtained by the slope of the log(*v*)–log(*i*). Generally, for the diffusion of the faradaic contribution, the *i* is linearly proportional to the square root of *v*; meanwhile, for the capacitive effects, *i* is proportional to the scan rate *v*.^[^ ^34^ ^]^ As shown in Figure S24a,b (Supporting Information), the *b* value is close to 0.5, indicating that the diffusion controlled by faradaic contribution is the mainly charge storage mechanism with or without the illumination. Meanwhile, the cycling performance of the battery assembled with a pure Au@N‐TiO_2_/CC electrode (without Li_2_S_6_) was evaluated at a current density of 167.5 mA g^−1^ (Figure S15b,c, Supporting Information). The test results show that very little increase in capacity was obtained after introducing light illumination, which also proves that the contribution of capacitance is almost negligible. In conclusion, these results show that photoinduced electron/hole accelerates sulfur redox reaction under light irradiation during charging and discharging process, which is also the main reason for capacity enhancement.

The cycling performance of the battery was conducted at a current density of 2 C (Figure 4j). The PALSB with Au@N‐TiO_2_/CC photoelectrode provides a high sulfur utilization with an ultra‐high discharge capacity of over 1200 mA h g^−1^ and keeps a reversible capacity of 879 mA h g^−1^ even after 40 cycles, which is much higher than PALSB without light illumination (256 mA h g^−1^). In addition, the electrode morphology was observed after 40 cycles (Figure S25a,b, Supporting Information). It can be seen that the discharge product Li_2_S was mostly decomposed with the illumination, which is in sharp contrast to the cathode morphology after cycling without illumination, further confirming that photogenerated holes effectively promote sulfur evolution reaction during the charge process. The electrochemical performance of PALSB with high sulfur load was also investigated to evaluate the application potential of Au@N‐TiO_2_/CC photoelectrode. Figure S26 (Supporting Information) shows the first GCD curves of Au@N‐TiO_2_/CC battery with high sulfur load (3.06 mg cm^‐2^) at 0.1 C with and without the illumination. The capacity of PALSB is improved, with an area capacity of 3.2 mA h cm^‐2^ with illumination, higher than that of 2.02 mA h cm^‐2^ without illumination, and remained stable in 15 cycles (Figure 4k), indicating the practical application potential of Au@N‐TiO_2_/CC as a multifunctional photocathode of PALSB. These results indicate that Au@N‐TiO_2_/CC photoelectrode inhibits the recombination of photogenerated electrons and holes, which can increase the concentration of carriers in the system and promote the absorption of visible light. Consequently, the utilization of active substances is effectively improved.

In addition, to avoid the photothermal effect of long‐band light on the performance of LSB, the solar simulator in this work is equipped with a 350–780 nm visible light filter, which can effectively remove infrared sunlight. Besides, the related experiments are supplemented to explore the effect of photothermal on the electrochemical performance of LSBs. The temperature sensor is exposed to a xenon lamp to record the temperature fluctuations. It was observed that the temperature increased by only 5.7 °C under light illumination in 90 min (Figure S27a–e, Supporting Information). The assembled LSB is placed under a light source, and the glass window is sealed with tin foil to ensure that it cannot transmit light but can transfer heat (Figure S27f, Supporting Information). As shown in Figure S28a (Supporting Information), the corresponding faradaic current increases of CV curves slightly while the peak of the redox position does not shift significantly when introduced the photothermal. Meanwhile, the interface contact impedance in the high‐frequency region and the charge transfer impedance in the mid‐frequency region is slightly decreased (Figure S28b, Supporting Information). These results show that the contribution of the photothermal effect to the electrochemical kinetic performance under illumination conditions is limited. To further investigate the contribution of photothermal effect to the discharge capacity of LSB, the capacity changes of Au@N‐TiO_2_/CC assembled batteries with the increase of temperature were tested at various current densities (Figure S28c, Supporting Information). When the temperature of the tested battery was increased by 5.7 °C, the average discharge capacity increased by 23, 38, 26 and 22 mA h g^−1^ at 0.5, 1, 2, and 3 C, respectively, which are much smaller than the contribution of photogenerated carriers to the capacity. Therefore, the photothermal effect had little effect on the electrochemical performance and the photogenerated carriers are the main factor in the improvement of electrochemical performance.^[^ ^35^ ^]^

### 2.4 Electrochemical Performance of PALSB

**Figure 5:**
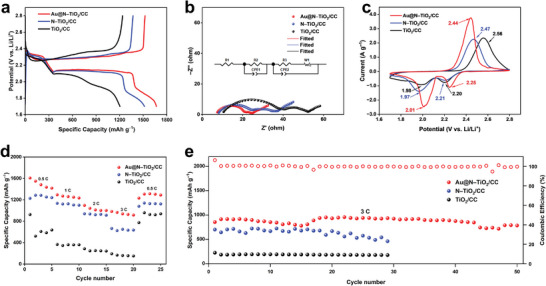
Electrochemical performance of PALSB with various photoelectrodes under illumination: a) GCD curves at 0.1 C, b) EIS curves, c) CV curves at 0.1 mV s^−1^, d) rate performance, e) cycling performance at 3 C.

To confirm the synergistic effect of Au and N to improve the catalytic property of TiO_2_, the electrochemical performance of PALSB assembled with Au@N‐TiO_2_/CC, N‐TiO_2/_CC, and TiO_2_/CC photoelectrodes was investigated. **Figure** **5**a shows the GCD curves of the batteries assembled with different photoelectrodes at 0.1 C. Compared with the batteries assembled with the other two cathodes, Au@N‐TiO_2_/CC battery has the lowest overpotential. The specific discharge capacity of Au@N‐TiO_2_/CC battery (1667 mA h g^−1^) is much higher than that of N‐TiO_2_/CC (1516 mA h g^−1^) battery and TiO_2_/CC (1204 mA h g^−1^) battery, indicating that the synergistic effect of Au and N promotes the redox reaction of sulfur under illumination. The Schottky barrier at the interface of Au and TiO_2_ leads to the effective separation of photogenerated electrons and holes, and the photogenerated electrons are transferred to Au, thereby reducing the recombination rate of electrons and holes, and improving the photocatalytic activity. The positive effect of the synergistic action of Au and N on the performance improvement of LSBs is also confirmed by EIS curves and CV curves. The Au@N‐TiO_2_/CC battery exhibits smaller interface contact resistance (*R*_2_) and charge transfer impedance (*R*_3_) compared with N‐TiO_2_/CC battery and TiO_2_/CC battery (Table S1, Supporting Information), indicating that photogenerated carriers can increase the charge transfer rate and improve the transport kinetics of electrons (Figure 5b). These results indicate the positive effect of the synergistic action of Au and N on the photocatalytic activity. The peaks at 2.21–2.25 and 2.01–1.97 V in the cathodic scan correspond to the reduction of S_8_ to soluble polysulfide and further conversion to Li_2_S. Meanwhile, the anodic peaks at 2.44–2.56 V represent the inverse transition of Li_2_S to S_8_. The reduction peak of Au@N‐TiO_2_/CC battery shows higher initial/peak voltages, while the oxidation peak shows lower initial/peak voltages, and the corresponding faradaic current also increased greatly, which is attributed to the efficient separation of photogenerated carriers, thereby accelerating the sulfur redox reaction (Figure 5c). The CV curves of TiO_2_/CC, N‐TiO_2_/CC and Au@N‐TiO_2_/CC assembled batteries under dark condition were also investigated. As shown in Figure S29 (Supporting Information), the results under dark are comparable to those observed in the light condition. It can be seen that the Au@N‐TiO_2_/CC battery has a smaller polarization voltage and stronger faraday response current, demonstrating that Au@N‐TiO_2_/CC has superior electrochemical catalytic performance, further proving that Au@N‐TiO_2_/CC electrode is a multifunctional catalyst with both electrocatalytic and photocatalytic performance. Meanwhile, the smaller Tafel slope of Au@N‐TiO_2_/CC photoelectrode under illumination also supports the above conclusion (Figure S30a–d, Supporting Information). The rate performance of Au@N‐TiO_2_/CC, N‐TiO_2_/CC and TiO_2_/CC batteries under illumination was evaluated at different current densities (0.5–3 C) (Figure 5d). Au@N‐TiO_2_/CC battery shows superior rate performance and provides a discharge specific capacity of 982 mA h g^−1^ at 3 C, which is much higher than that of N‐TiO_2_/CC battery (664 mA h g^−1^) and TiO_2_/CC battery (192 mA h g^−1^). Cycling performance of Au@N‐TiO_2_/CC, N‐TiO_2_/CC, and TiO_2_/CC batteries was evaluated at a high current of 3 C under light illumination (Figure 5e). The specific discharge capacity of N‐TiO_2_/CC battery is only 702 mA h g^−1^ in the first cycle, and rapidly decreases to 462 mA h g^−1^ after about 30 cycles. The catalytic activity of TiO_2_/CC battery is poor, with a maximum discharge capacity of only 225 mA h g^−1^ at 3 C. On the contrary, the discharge capacity of the Au@N‐TiO_2_/CC battery reaches 855 mA h g^−1^ in the first cycle and the capacity retention rate is 92% after 50 cycles, indicating a good cycle stability of PALSB.

Furthermore, to verify the photocharging performance of Au@N‐TiO_2_/CC battery, the PALSB after discharge is only charged by light at the window side without applying any external voltage. Meanwhile, the solar energy conversion efficiency (*η*_SCE_) of Au@N‐TiO_2_/CC assembled PALSB was calculated by the formula of *η*_SCE_ = *E*_out_/(*P*_in_×*t*×*A*) × 100%, where *E*_out_ is the discharge energy, *P*_in_ stands for the light power density, *t* refers to the photocharging time, and *A* represents the active photocathode area. Encouragingly, the PALSB exhibits a discharge capacity of 101 mA h g^−1^ with a maximum *η*_SCE_ of 0.99% after only 1 h of photocharging at 0.2 C. Moreover, the PALSB can work steadily for 7 cycles with low capacity fading under the condition of only photo charge, indicating that the Au@N‐TiO_2_/CC cathode is able to realize the integration of photoelectric conversion and energy storage. In addition, the capacity contribution of photothermal and polarization effects of PALSB are excluded (Figure S31a, Supporting Information). To explore the maximum capacity that photo charging can achieve, the charge time is gradually extended from 1 to 15 h. It can be seen that after photocharging for 5 h, the increase in capacity is very slow. Even if the charging time is extended to 15 h, its capacity can only be increased to 186 mA h g^−1^, indicating that photogenerated holes just can oxidize Li_2_S to polysulfides rather than to S_8_ (Figure S31b, Supporting Information). As shown in Figure S32 (Supporting Information), the voltage of Au@N‐TiO_2_/CC battery reaches 2.2 V after 5 h of photocharging, and provides a discharge specific capacity of 179 mA h g^−1^ at 0.2 C, reaching the level of traditional lithium‐ion batteries.^[^ ^36^ ^]^ To analyze the principle of bare photocharging, the conversion of Li_2_S to S_8_ at different photo charging stages was studied by Raman and XPS analysis. As shown in Figure S33a (Supporting Information), in the initial phase of photo charging, the vibrational peak of Li_2_S at 363 cm^−1^ can be clearly observed, while it disappears after 5 h of photocharging. Meanwhile, the vibrational peak at about 403 cm^−1^ assigned to polysulfides (Li_2_S*_x_*, 2<*x*<8) can be detected.^[^ ^37^ ^]^ The test results indicate the conversion of Li_2_S to polysulfides rather than to S_8_ within 5 h of photocharging. Similar results are obtained by XPS analysis, where the characteristic peak of Li_2_S disappears while the peak intensity of polysulfides increases from 9.6% to 14.1%, and no characteristic peaks of S_8_ can be observed (Figure S33b,c, Supporting Information). Table S2 (Supporting Information) summarizes the electrochemical performance of the state‐of‐the‐art photoelectrodes used for photoassisted/photocharged LSBs. It can be noticed that Au@N‐TiO_2_/CC has certain advantages in capacity and cycle performance when used as a photoelectrode of PALSB.

## 3 Conclusion

In summary, a freestanding photoelectrode fabricated by Au@N‐TiO_2_/CC heterostructure was developed for PALSB. The TiO_2_ nanorods possess outstanding photon capture ability while the doping of N element and the decoration of Au nanoparticles can further reduce the bandgap, enhancing the absorption of visible light and increasing the carrier separation rate, which improves the electrochemical performance of LSBs through photocatalytic effect and photoconductive effect. Photogenerated electrons accelerate the conversion of sulfur to insoluble Li_2_S, and photogenerated holes promote the reverse reaction during charging. Meanwhile, the Au@N‐TiO_2_/CC also can be employed as an electrocatalyst to accelerate the conversion of intermediate polysulfides during the charge/discharge process, further improving the electrochemical reaction kinetics. Attributed to the synergistic effect of electrocatalysis and photocatalysis, the PALSB with Au@N‐TiO_2_/CC photoelectrode not only achieves an ultrahigh specific capacity (1667 mA h g^−1^) with an excellent rate performance (982 mA h g^−1^ at 3 C), but also realizes outstanding cycling performance in the both cases of conventional and high sulfur loadings. In addition, the Au@N‐TiO_2_/CC assembled PALSB can be directly charged under light illumination without applying external circuit voltage, obtaining a discharge specific capacity of 179 mA h g^−1^, reaching the level of conventional lithium‐ion batteries. This work presents a novel approach to incorporating solar energy into the second battery system, which not only offers a new strategy for developing multifunctional photoelectrode for LSBs, but also expands the application of solar energy.

## 4 Experimental Section

Detailed experimental procedures can be found in the Supporting Information.

## Conflict of Interest

The authors declare no conflict of interest.

## Author Contributions

F.Z. and K.Y. contributed equally to this work. F.Z.: formal analysis, data curation, writing‐original draft. K.Y.: methodology, software. Y.L.: Uv–vis absorption spectra test. J.L.: investigation, formal analysis. C.L.: data curation. X.X.: formal analysis. Y.H.: writing‐review & editing, conceptualization, and supervision.

## Supporting information

Supporting Information

## Data Availability

The data that support the findings of this study are available from the corresponding author upon reasonable request.
