# Supplementary material for: Developing a Multifunctional Cathode for Photoassisted Lithium–Sulfur Battery
Source: Adv Sci (Weinh). 2024 Jul 19;11(35):2402978. doi: 10.1002/advs.202402978 (PMC11425247; doi:10.1002/advs.202402978)
Supplement: Supplementary file 1 — Supporting Information [file ADVS-11-2402978-s001.docx]

**Supporting Information**

**Developing A Multifunctional Cathode for Photo–Assisted Lithium–Sulfur Battery**

*Fei Zhao, Ke Yang,* *Yuxin Liu, Juan Li, Chan Li, Xinwu Xu, and Yibo He**

State Key Laboratory of Solidification Processing, Center of Advanced Lubrication and Seal Materials, School of Materials Science and Engineering, Northwestern Polytechnical University, Xi’an, Shaanxi, 710072, P. R. China

E–mail: heyibo@nwpu.edu.cn (Y. He)

**Experimental Section**

**Materials**

Carbon cloth (WOS1011) was purchased from CeTech (Taiwan). Titanium butoxide (C_16_H_36_O_4_Ti, 99.0%), isopropanol (99.7%), acetone (99.5%), ethanol (99.7%) and hydrochloric acid (36–38%) were purchased from Sinopharm Chemical Reagent. Chloroauric acid (HAuCl_4_·*x*H_2_O, ≥47.5%), Melamine, Tetraethylene glycol dimethyl ether, Lithium sulfide and sulfur powder were purchased from Aladdin (China).

**Preparation of TiO_2_/CC**

The carbon cloth (CC) was cut into electrodes with a diameter of 10 mm, and ultrasonically washed with acetone, ethanol, and deionized water for several times, then dried at 60 °C in a vacuum oven. The cleaned CC was immersed in 75 mM titanium butoxide/isopropanol solution for 10 minutes and dried in the oven. After repeating this process for 3 times, the sample was annealed at 500 °C for 1 h. 0.66 mL of titanium butoxide was added to 30 ml of 6 M hydrochloric acid and stirred for 6 h. Subsequently, the annealed CC was immersed in the above mixed solution and hydrothermal at 150 °C for 10 h. Finally, the CC was rinsed with deionized water and dried for use.

**Preparation of N–TiO_2_/CC**

The as prepared TiO_2_/CC was putted on a piece of carbon paper and then covered on a porcelain boat loaded with melamine powder, and calcinated at 750 °C for 2 h in Ar atmosphere.

**Preparation of** **Au@N–TiO_2_/CC**

Based on the previously reported, ^[S1]^ appropriate amount of 100 mM chloroauric acid/ethanol solution was added to a tiny quartz boat as the precursor of Au. The prepared N–TiO_2_/CC was put into another quartz boat, placed it in the tube furnace with a N_2_ gas flow. The tube furnace was heated up to 500 °C with an increase rate of 35 °C/min, and was kept at 500 °C for 2 h.

**Preparation of** **Li_2_S_6_**

The sulfur powder and lithium sulfide (molar ratio of 5:1) were dissolved in the prepared lithium–sulfur electrolyte (1 M LiTFSI was dissolved in DME and DOL (v/v=1:1) with 2 wt% LiNO_3_), red–brown Li_2_S_6_ solution was obtained after stirring at 70 °C for 48 h.

**Preparation of** **Li_2_S_8_**

The sulfur powder and lithium sulfide (molar ratio of 5:1) were dissolved in 1 M LiTFSI/ tetraethylene glycol dimethyl ether solution, dark–brown Li_2_S_8_ solution was obtained after stirring at 70 °C for 24 h.

**Assembly of PALSB**

PALSB was assembled in an argon–filled glove box with water and oxygen levels below 0.01 ppm. Commercial Celgard 2325 and Li metal foils were used as separators and anodes, respectively. The electrolyte is 1 M LiTFSI dissolved in DME and DOL (v/v=1:1) with 2 wt% LiNO_3_. The sulfur loading is fixed at about 1 mg cm^–2^ in the form of 0.5 M Li_2_S_6_ solution dropped on the surface of the cathode. The positive case with a glass window was sealed with epoxy resin glue to assemble 2032 type coin cells.

**The nucleation test of Li_2_S**

12 μL of 0.25 M Li_2_S_8_ solution was added to the cathode side, and the anode side was the normal lithium–sulfur electrolyte. The cell was discharged at a constant current of 0.112 mA and then discharged at a constant voltage for 24000 s.

**Characterization**

The chemical compositions of the TiO_2_/CC, N–TiO_2_/CC and Au@N–TiO_2_/CC were characterized by X–ray diffraction using Cu–Kα radiation, λ=1.5418 Å (XRD, D8 ADVANCE BRUKER), microconfocal Raman spectrometer (Alpha 300R), and X–ray photoelectron spectrometer (XPS, PHI 5000 VersaProbe III). The morphologies of the materials were characterized by scanning electron microscope (SEM, Tescan Clara GMH ) and transmission electron microscope (TEM, Talos F200X). Photoluminescence emission spectra (PL, Hitachi–F4600) were measured to analyze the fate of photo–generated electron–hole pairs.

**Electrochemical Measurements**

The cycling performance of the electrodes was evaluated on a Neware battery test system, and cyclic voltammograms (CV) curves and electrochemical impedance spectra (EIS) were obtained on a CHI660E electrochemical workstation. Mott–Schottky plots was measured according to a three–electrode system in 0.5 M Na_2_SO_4_ solution (PH=7.0), with a Pt foil counter–electrode and Ag/AgCl reference electrode. A 300 W Xe lamp was used as the light source, and the power was fixed at 60 mW cm^–2^.

**The carrier density was calculated:**

|  | N_d_ = (2/qεε_0_)/[d(1/C^2^)/dV] | (S1) |
| --- | --- | --- |

where, ε and ε_0_ are the dielectric constant of the semiconductor and the permittivity of the vacuum, respectively. q stands for the electron charge, N_d_ is the donor density, and d(1/C^2^)/dV represents the slope of the M–S plots.

**Lithium ions diffusion coefficient**

*Randles–Sevcik* equation:

|  | I_p_=2.69×10^5^**·**n^3/2^**·**S·$\text{D}_{\text{Li}}^{\text{1/2}}$**·**C_Li_**·**v^1/2^ | (S2) |
| --- | --- | --- |

where, I_p_ is the peak current, n represents the number of transferred electrons (n=2 in LSBs), S stands for the area of the test electrode, D_Li_ corresponds to the diffusion coefficient of Li^+^, v represents the scanning rate, and C_Li_ refers to the concentration of Li^+^ involved in redox in the system.

**Supplementary Figures and Tables**


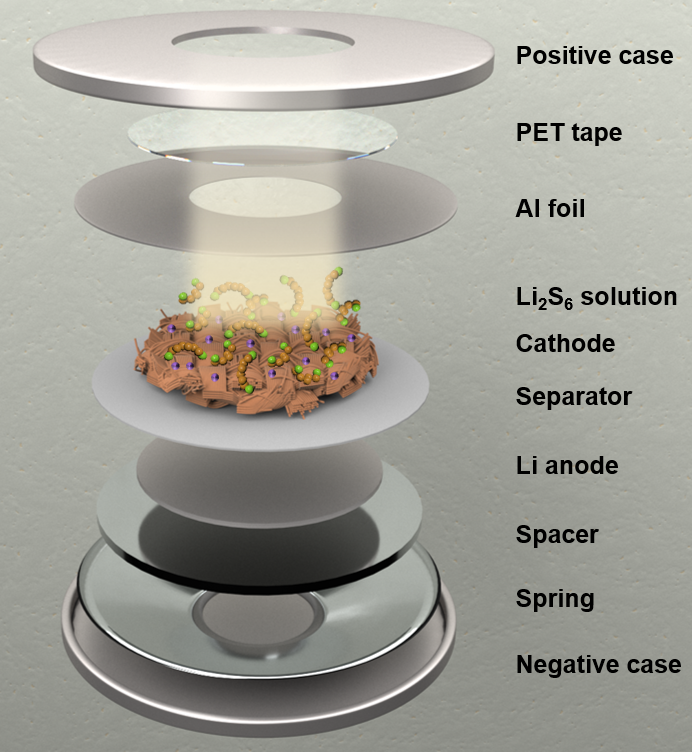


Figure S1. Schematic assembly of PALSB.


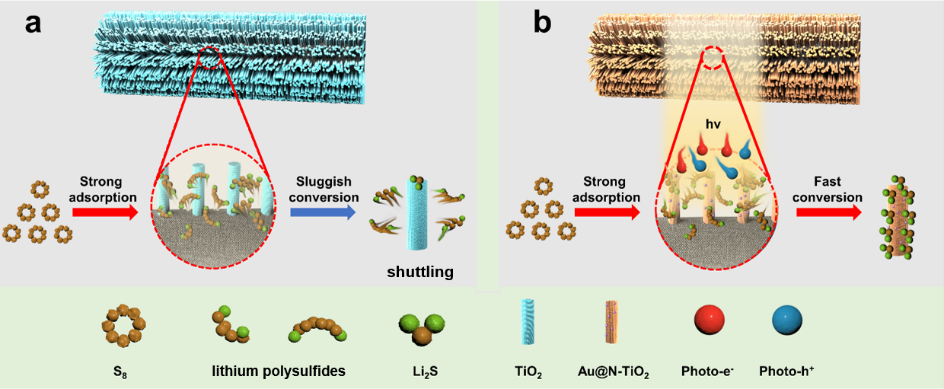


Figure S2. Schematic of a) TiO_2_ and b) Au@N–TiO_2_ catalysis for lithium polysulfides.


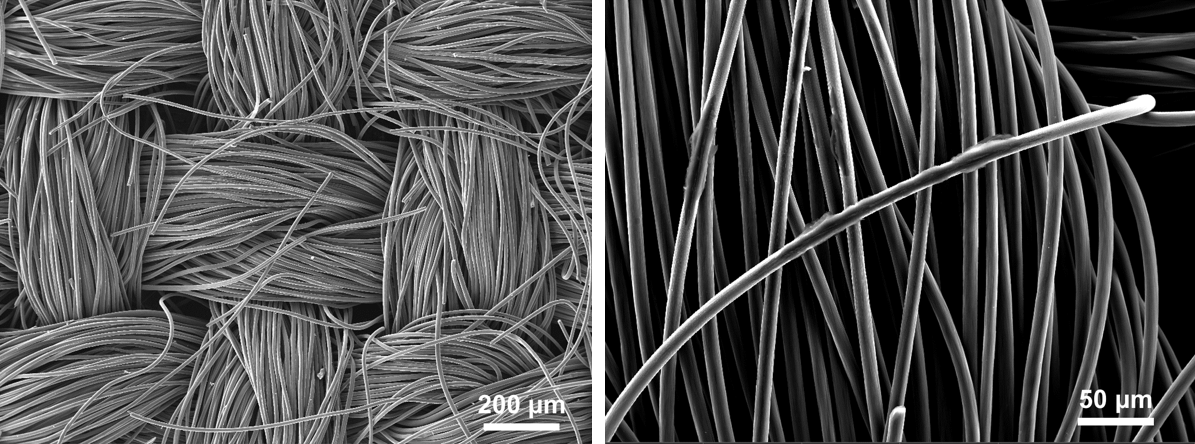


Figure S3. Low–magnification SEM image of CC.


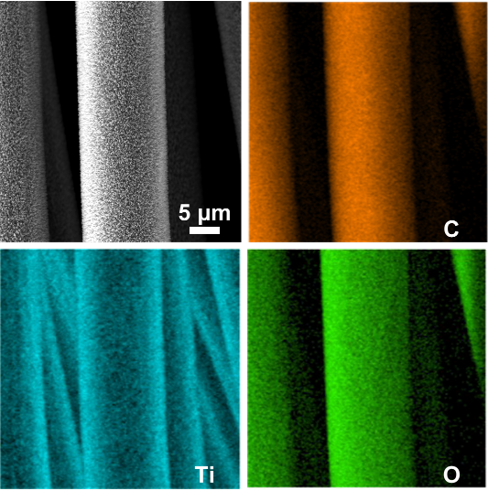


Figure S4. Elemental mapping image of TiO_2_/CC.


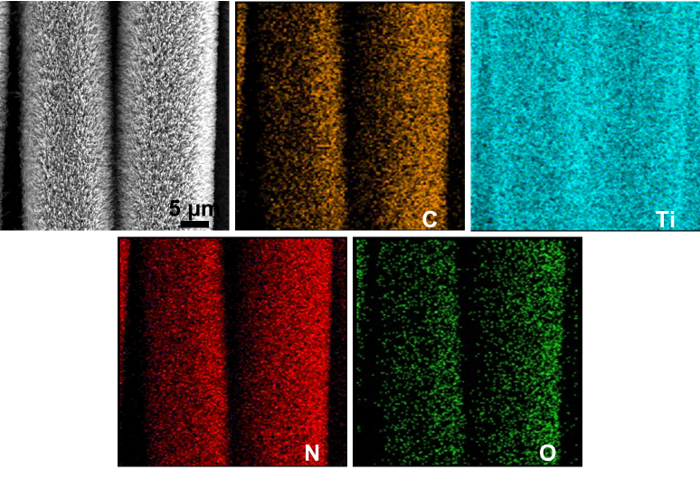


Figure S5. Elemental mapping image of N–TiO_2_/CC.


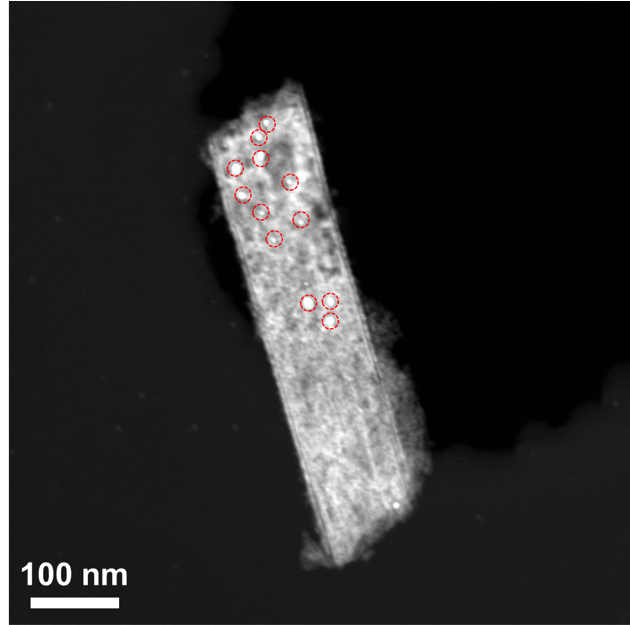


Figure S6. HAADF image of Au@N–TiO_2_/CC.


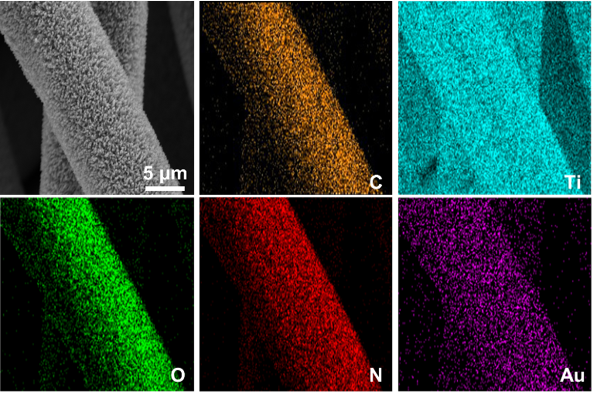


Figure S7. Elemental mapping image of Au@N–TiO_2_/CC.


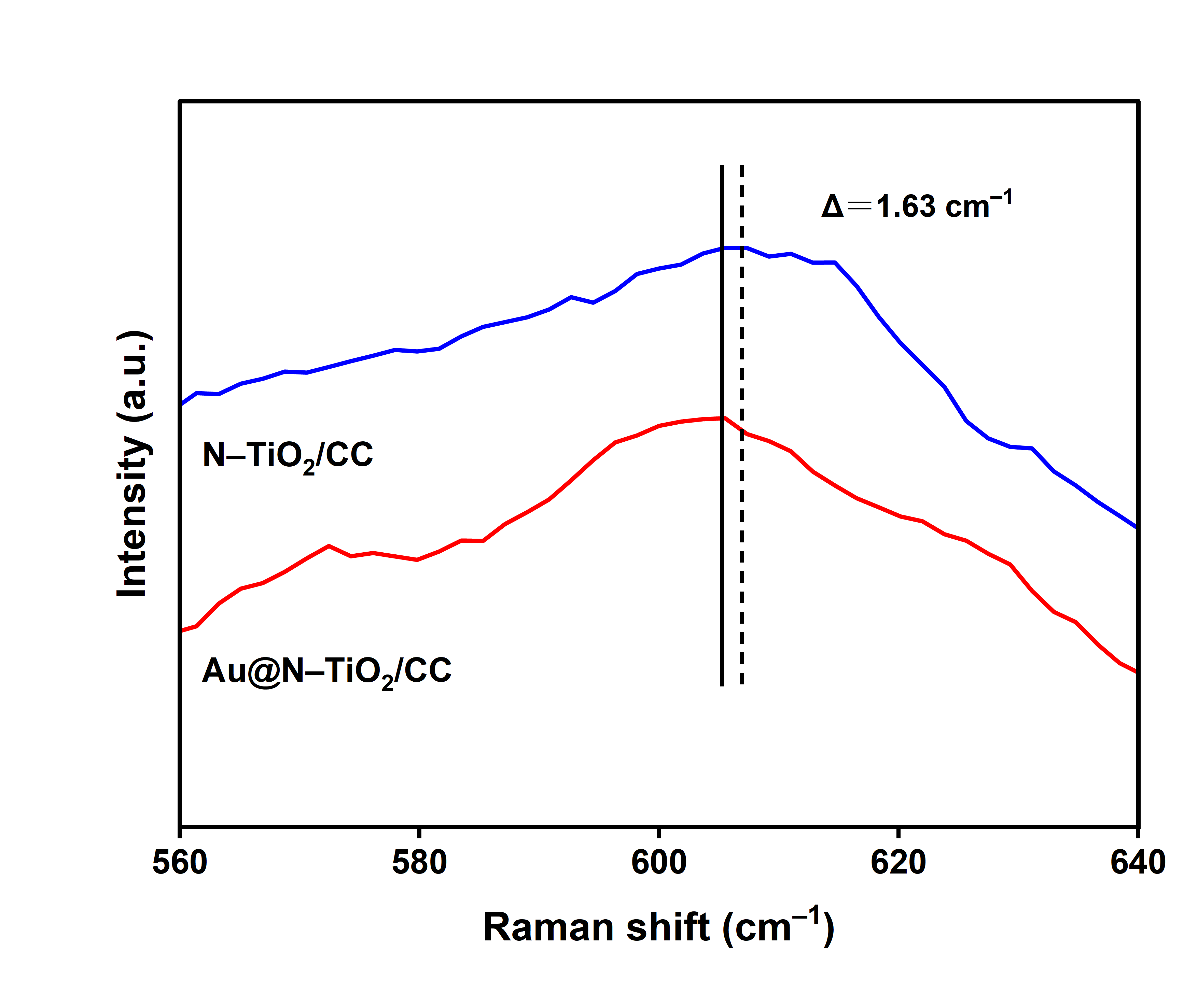


Figure S8. Raman shift of N–TiO_2_/CC and Au@N–TiO_2_/CC.


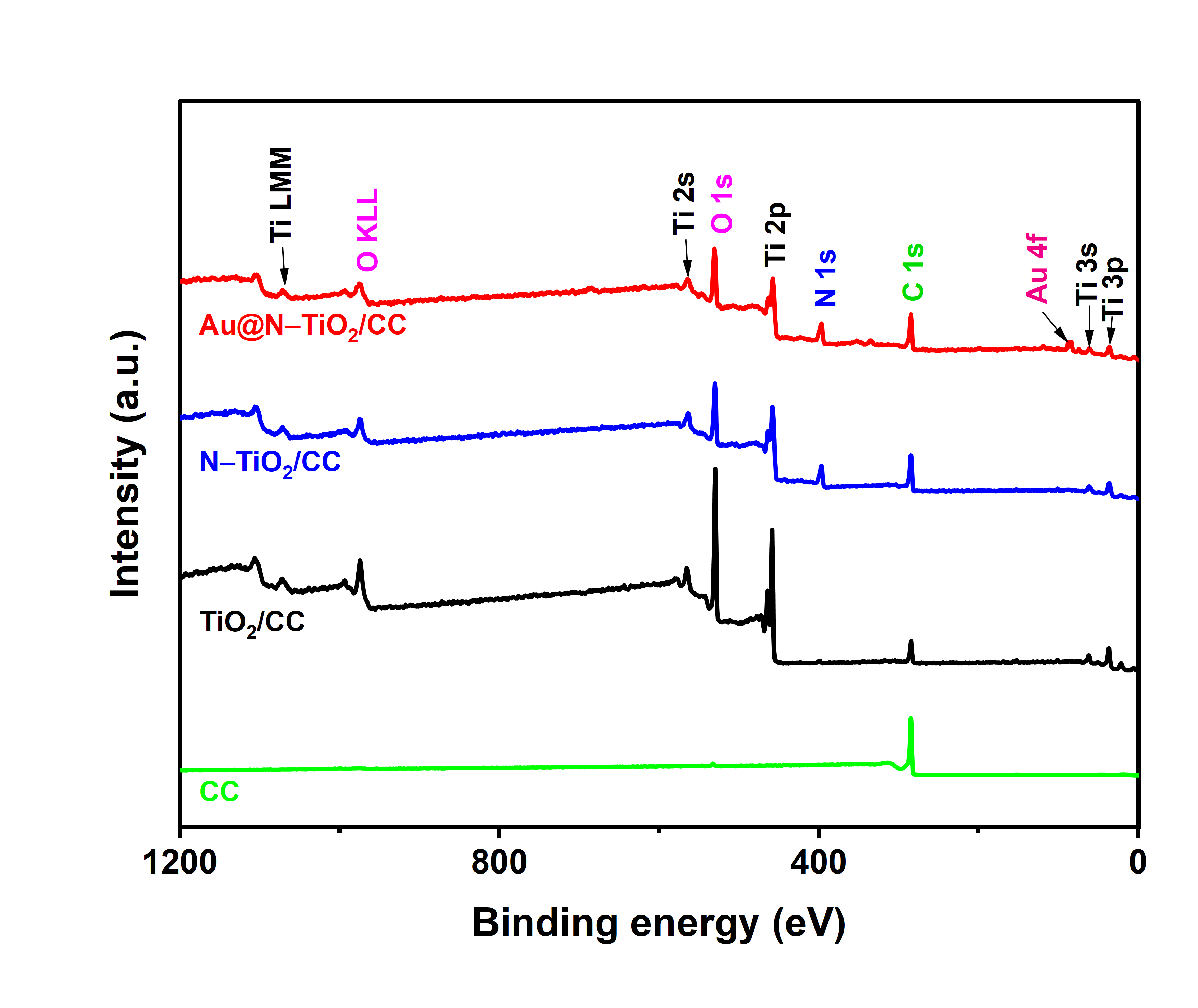


Figure S9. XPS full spectra of CC, TiO_2_/CC, N–TiO_2_/CC and Au@N–TiO_2_/CC.


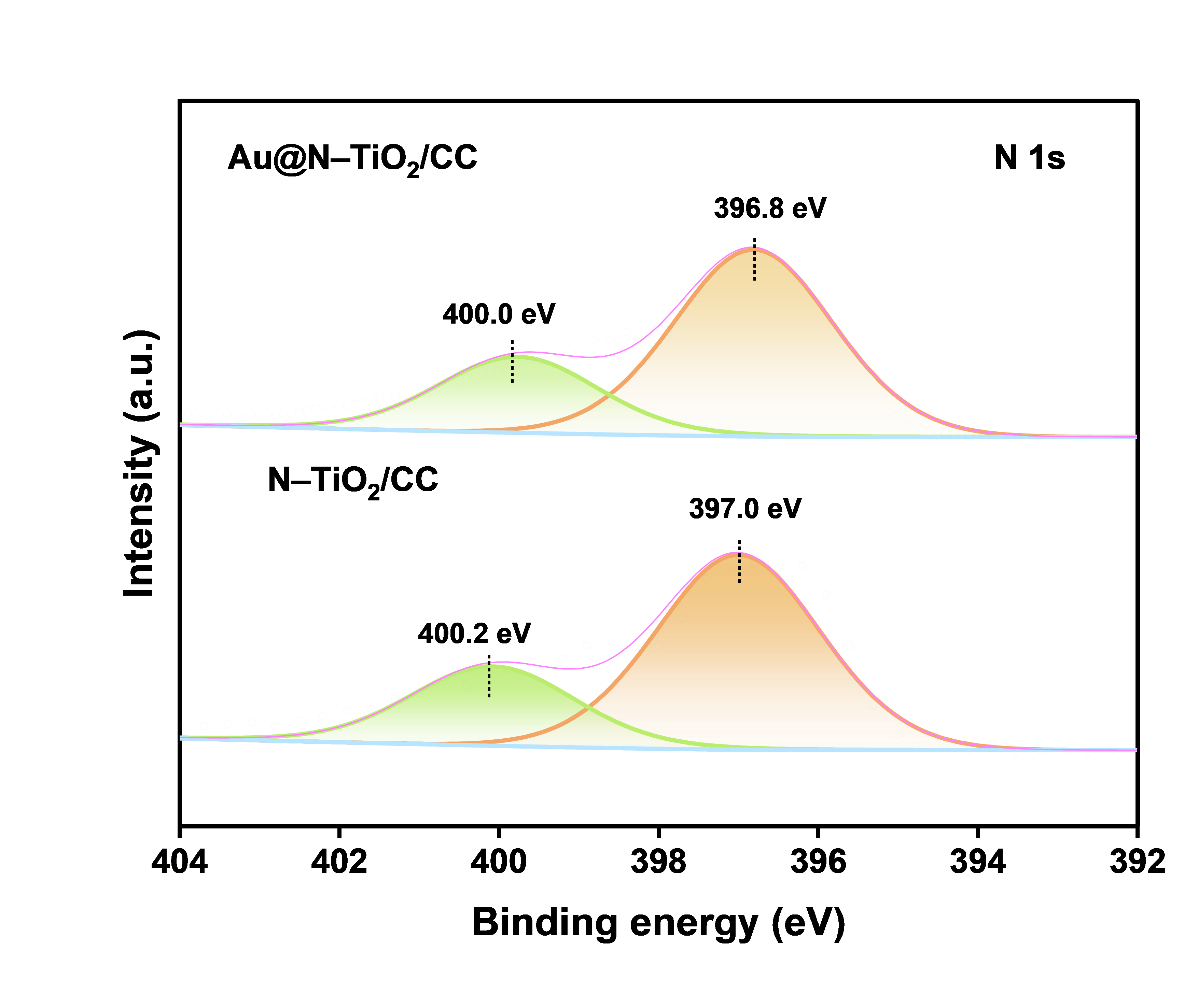


Figure S10. N 1s XPS spectra of N–TiO_2_/CC and Au@N–TiO_2_/CC.


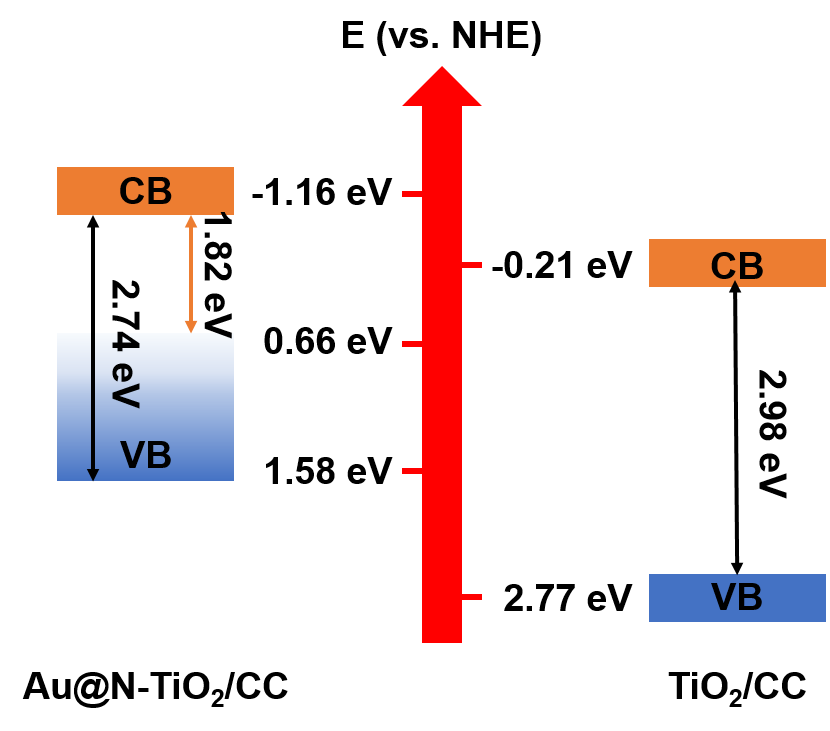


Figure S11. Band diagram of TiO_2_/CC and Au@N–TiO_2_/CC.


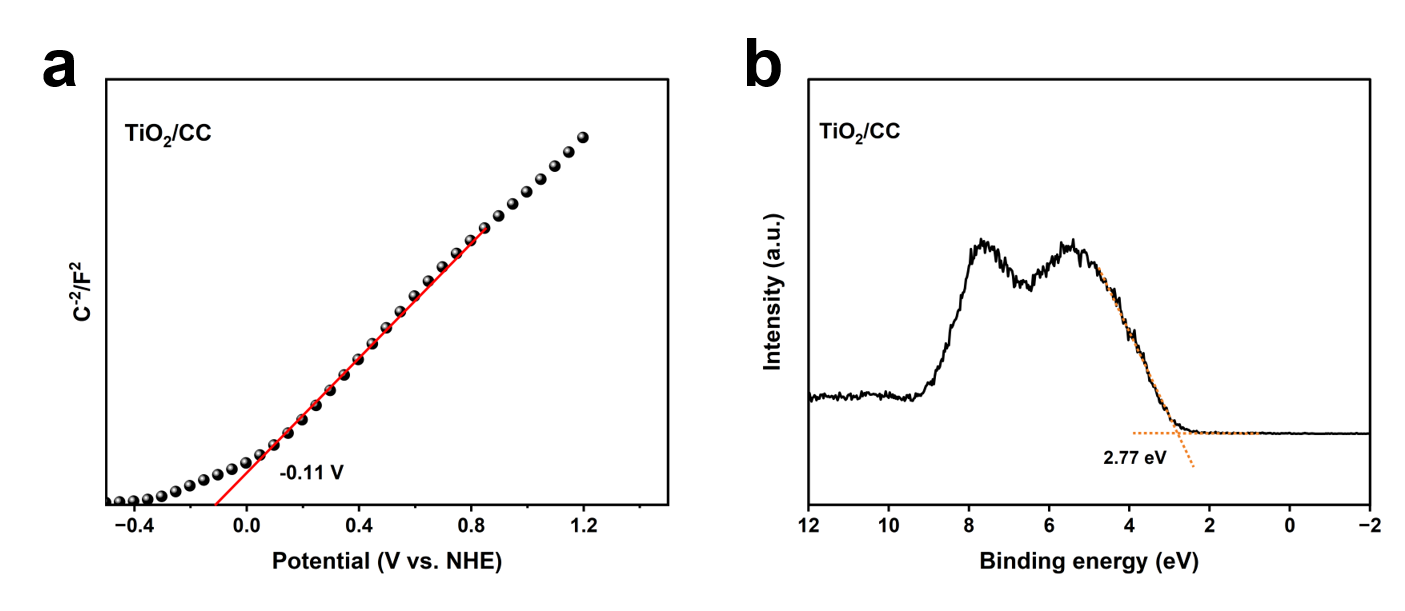
Figure S12. a) Mott–Schottky plot of TiO_2_/CC, b) XPS valence band spectra of TiO_2_/CC.


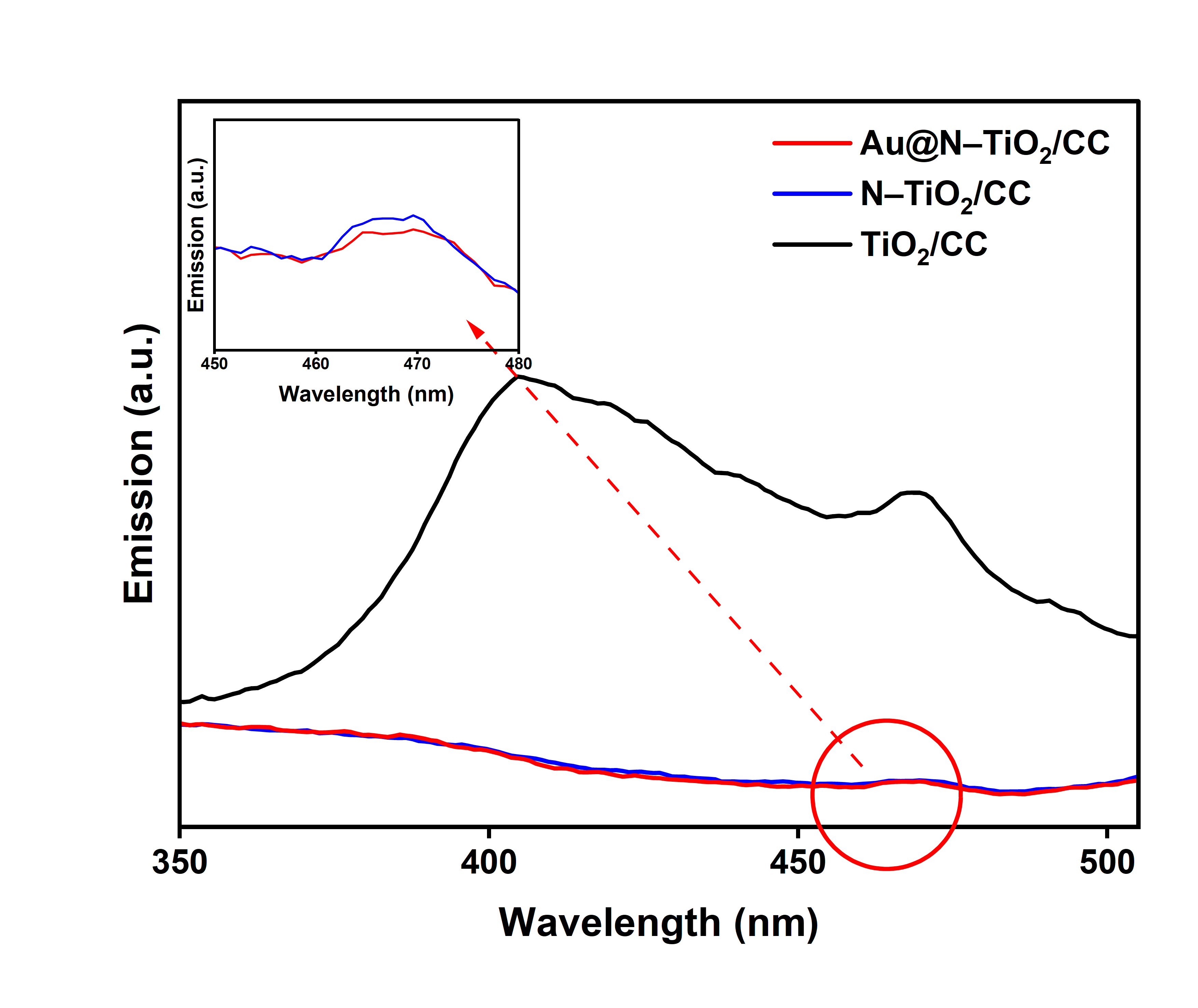


Figure S13. PL emission spectra of TiO_2_/CC, N–TiO_2_/CC and Au@N–TiO_2_/CC.


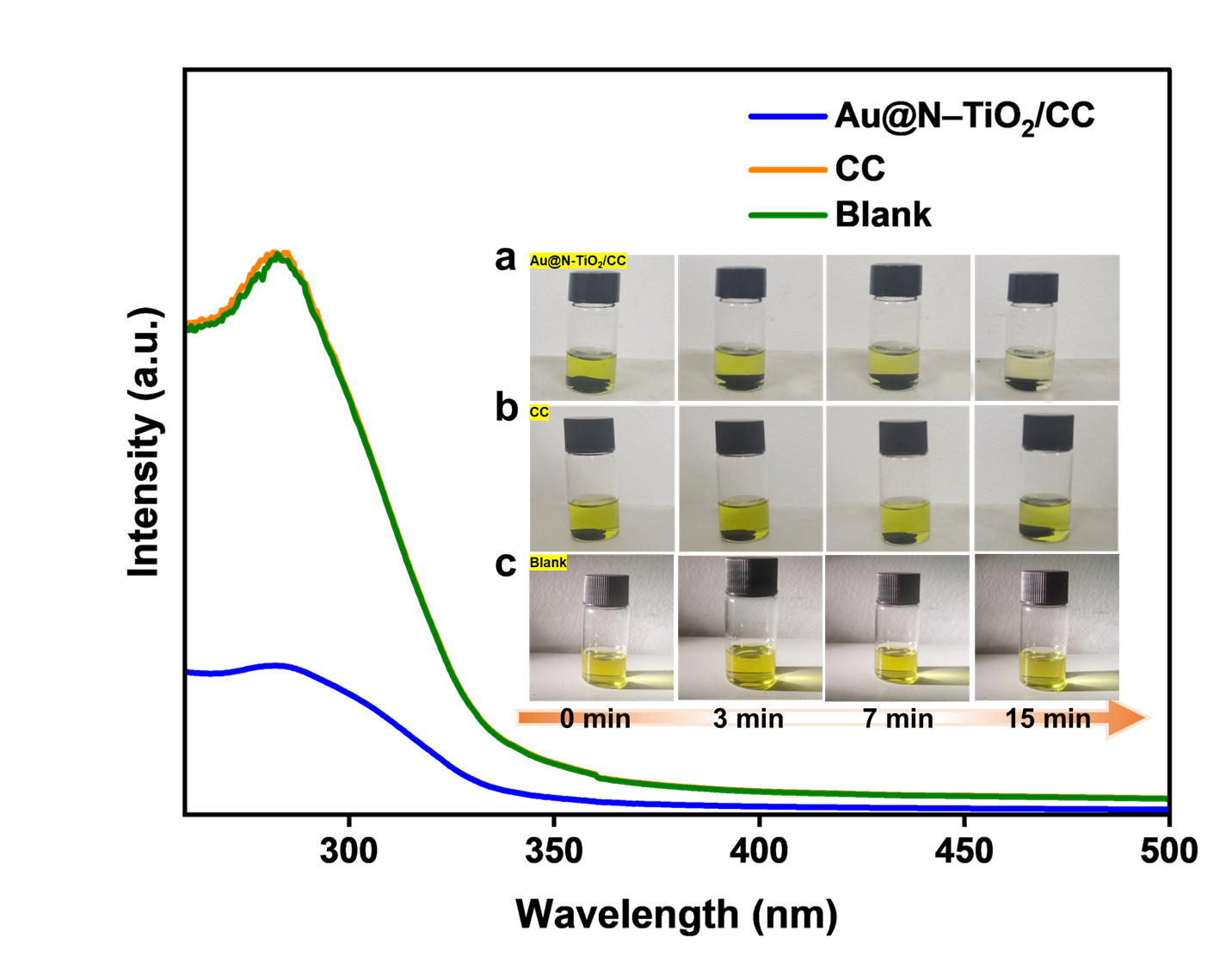


Figure S14. UV–vis absorption spectra of Li_2_S_6_ solution with different samples (inset: the process of visual adsorption of Li_2_S_6_ solution).


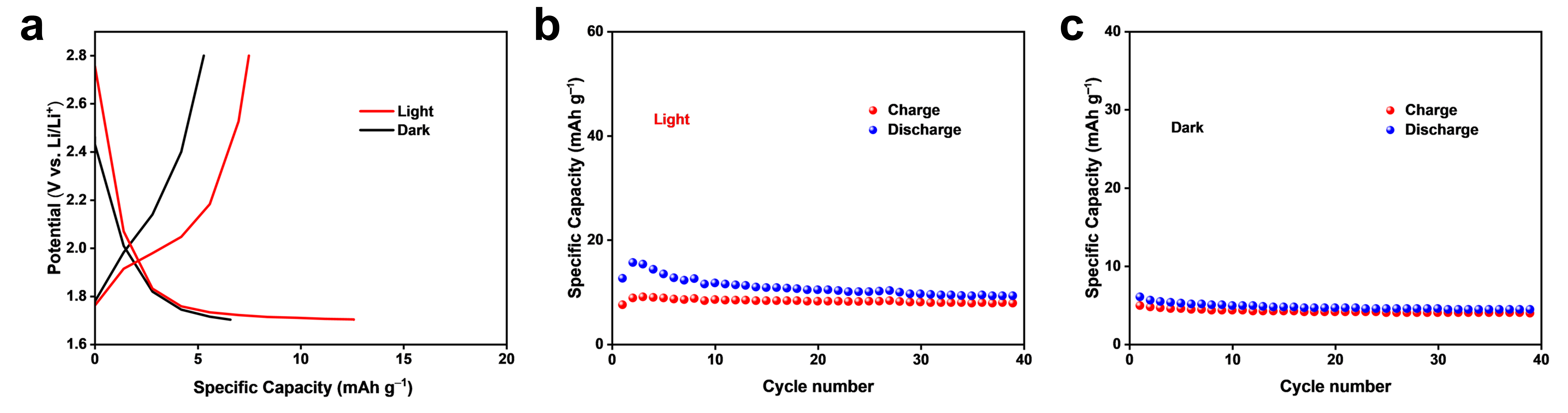


Figure S15. The electrochemical performance of the battery assembled by an Au@N–TiO_2_ cathode without Li_2_S_6_: a) GCD curves; cycling performance at a current density of 0.1 C b) with and c) without the light illumination.


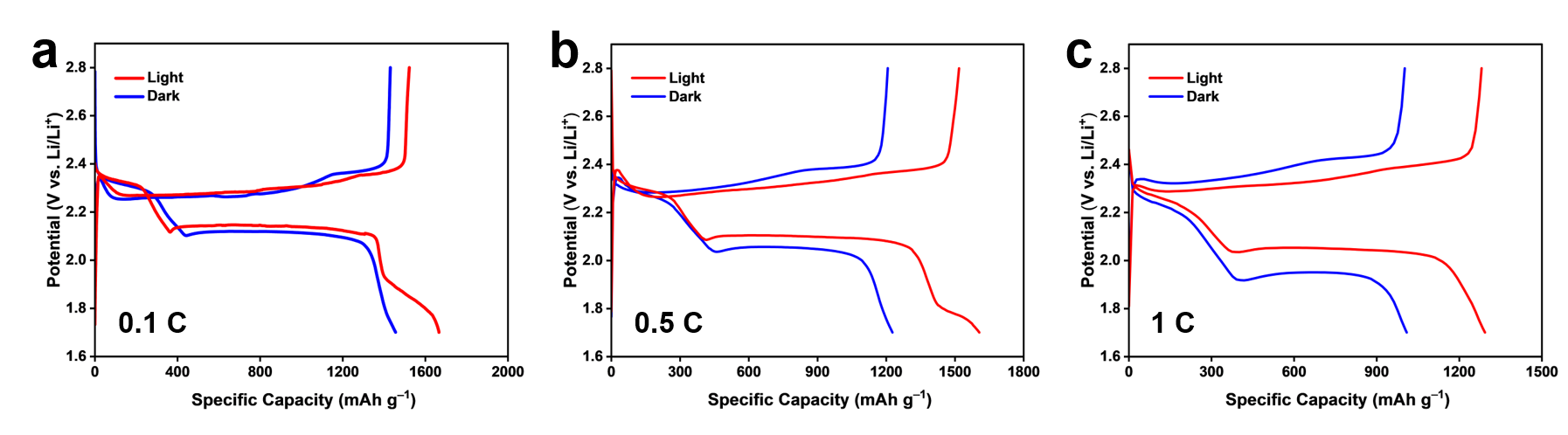


Figure S16. GCD curves of Au@N–TiO_2_/CC battery with and without the light illumination at a) 0.1 C; b) 0.5 C and c) 1 C.


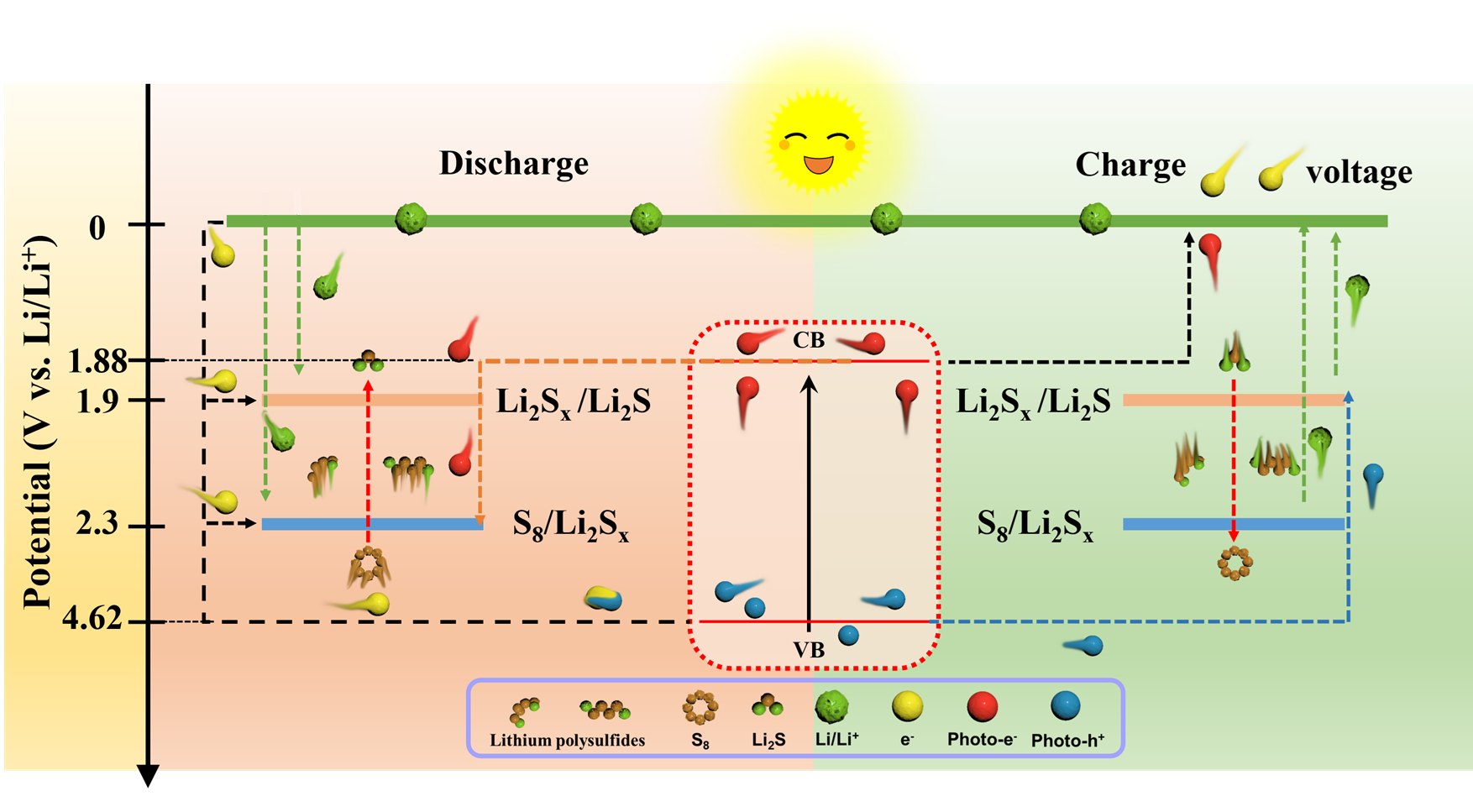


Figure S17. Band energy diagram of Au@N**–**TiO_2_/CC and Li_2_S/S_8_ vs. Li/Li^+^.


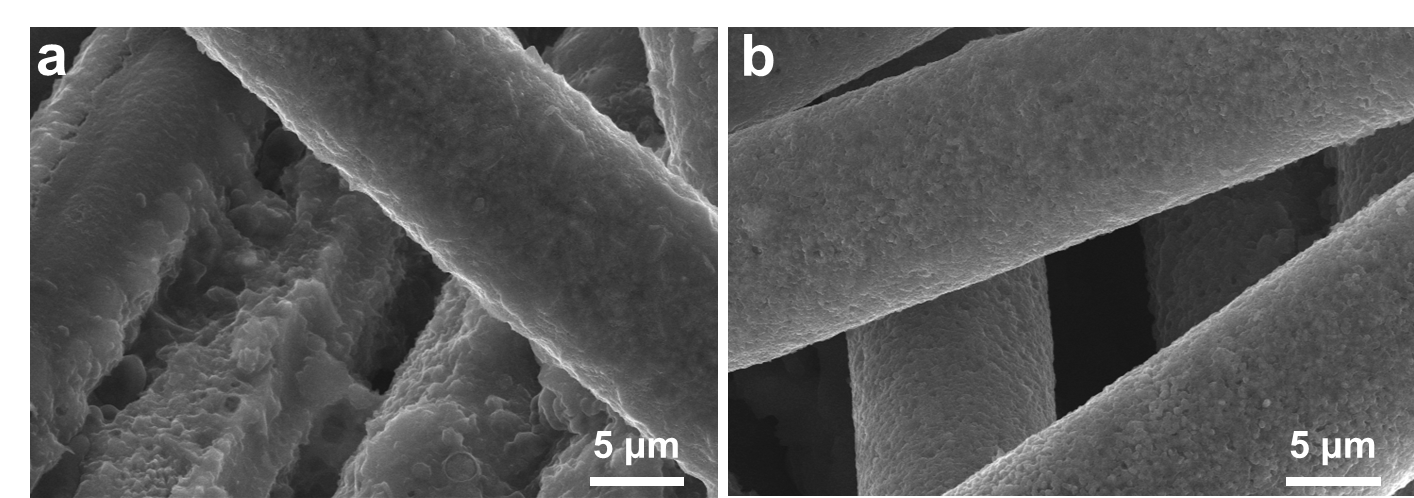


Figure S18. The SEM images of Au@N–TiO_2_/CC electrodes after first discharge a) without and b) with the illumination.


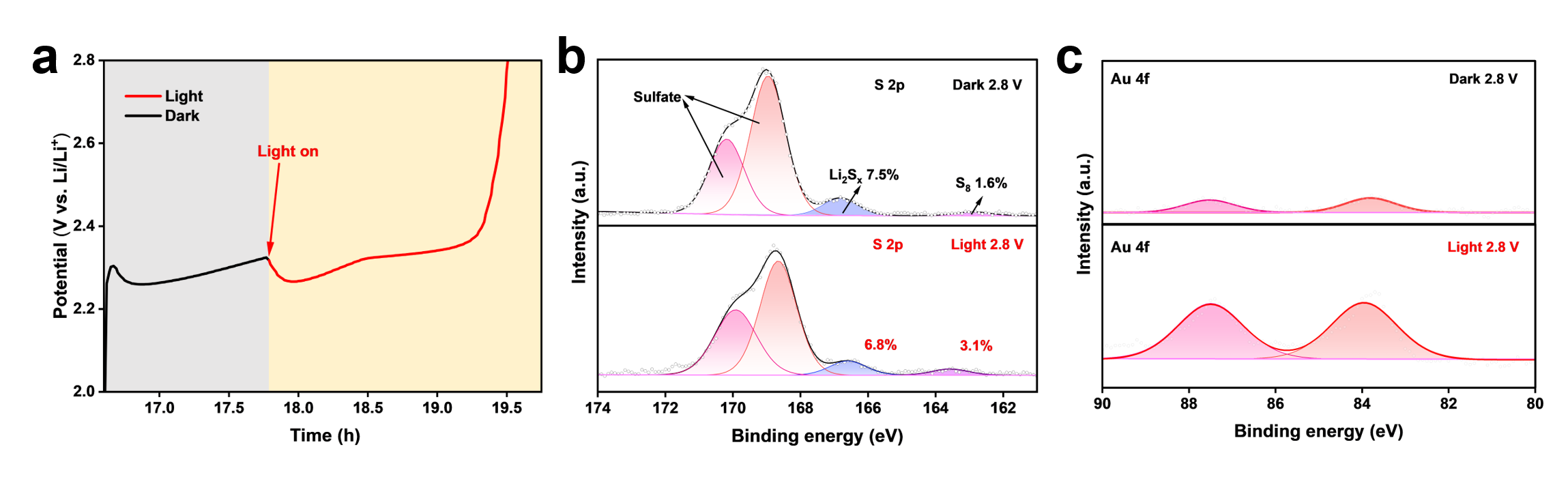


Figure S19. a) Galvanostatic charge curve at 0.2 C. High resolution XPS spectra of b) S 2p and c) Au 4f of Au@N–TiO_2_/CC electrodes after charging to 2.8 V with and without the illumination.


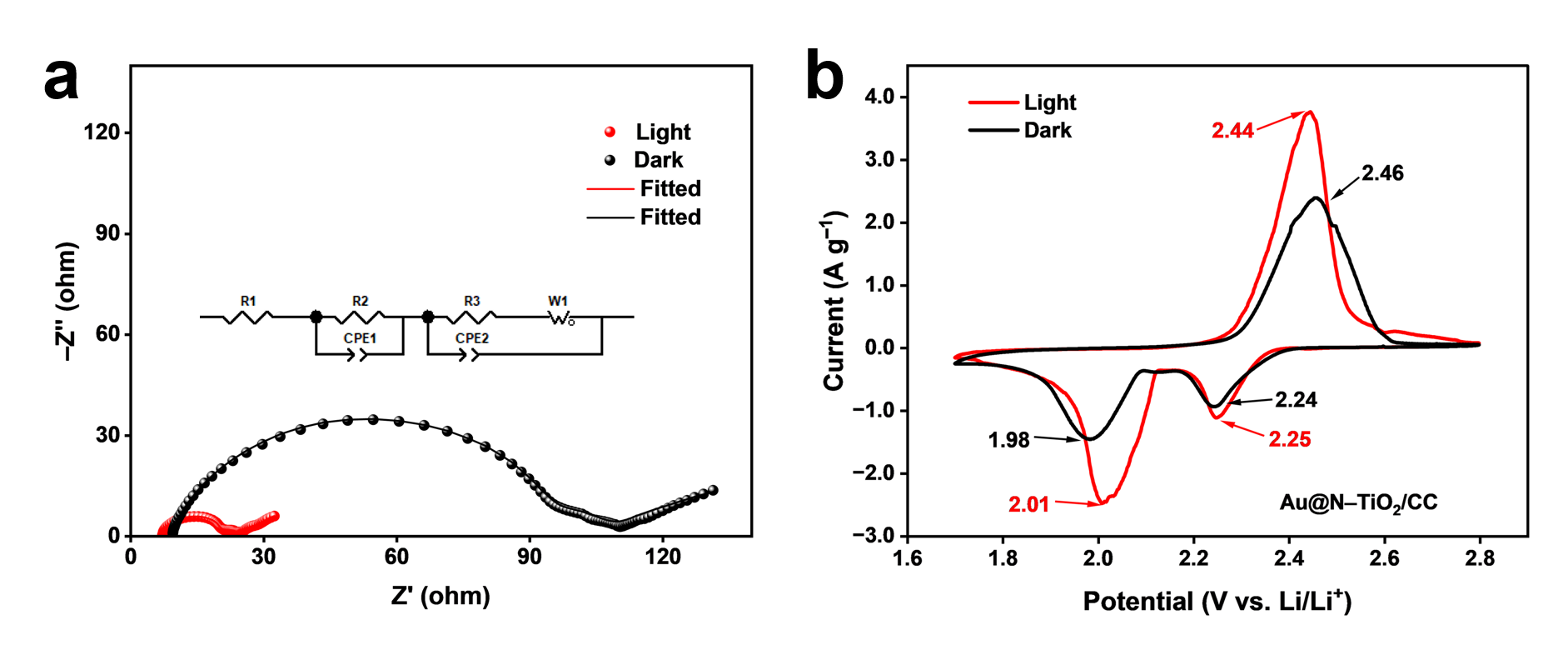


Figure S20. a) EIS curves, b) first cycle of CV curves at 0.1 mV s^–1^ of Au@N–TiO_2_/CC battery with and without the light illumination.


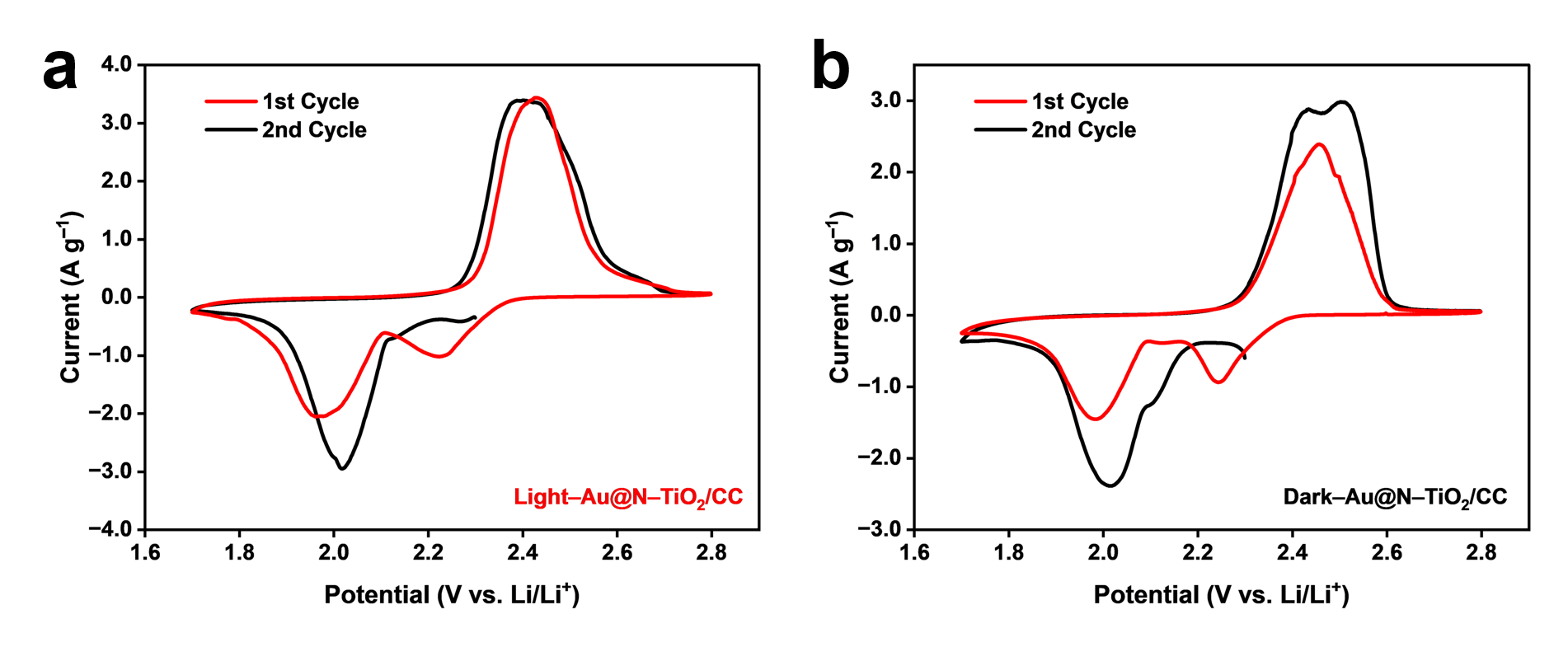


Figure S21. The initial two cycles of CV curves of the Au@N–TiO_2_/CC battery a) with and b) without the light illumination.


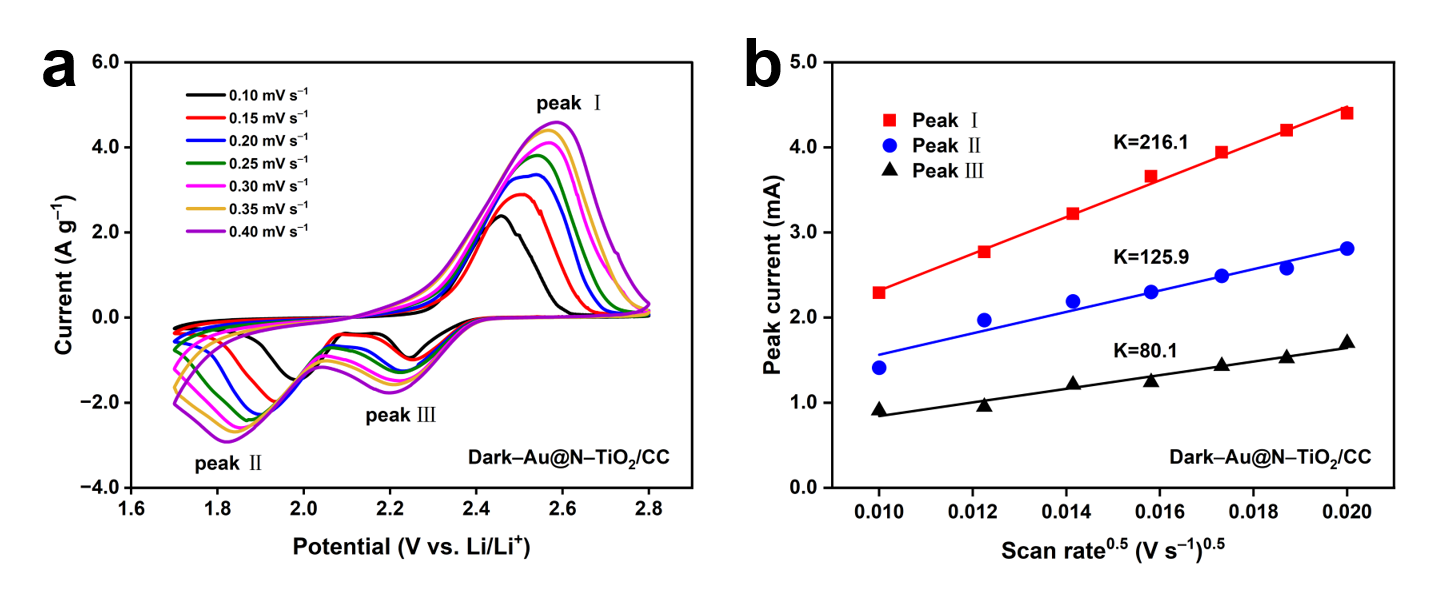


Figure S22. a) CV curves of Au@N–TiO_2_/CC battery without illumination at different sweep rates. b) Plots of CV peak current vs the square root of the scan rate without illumination.


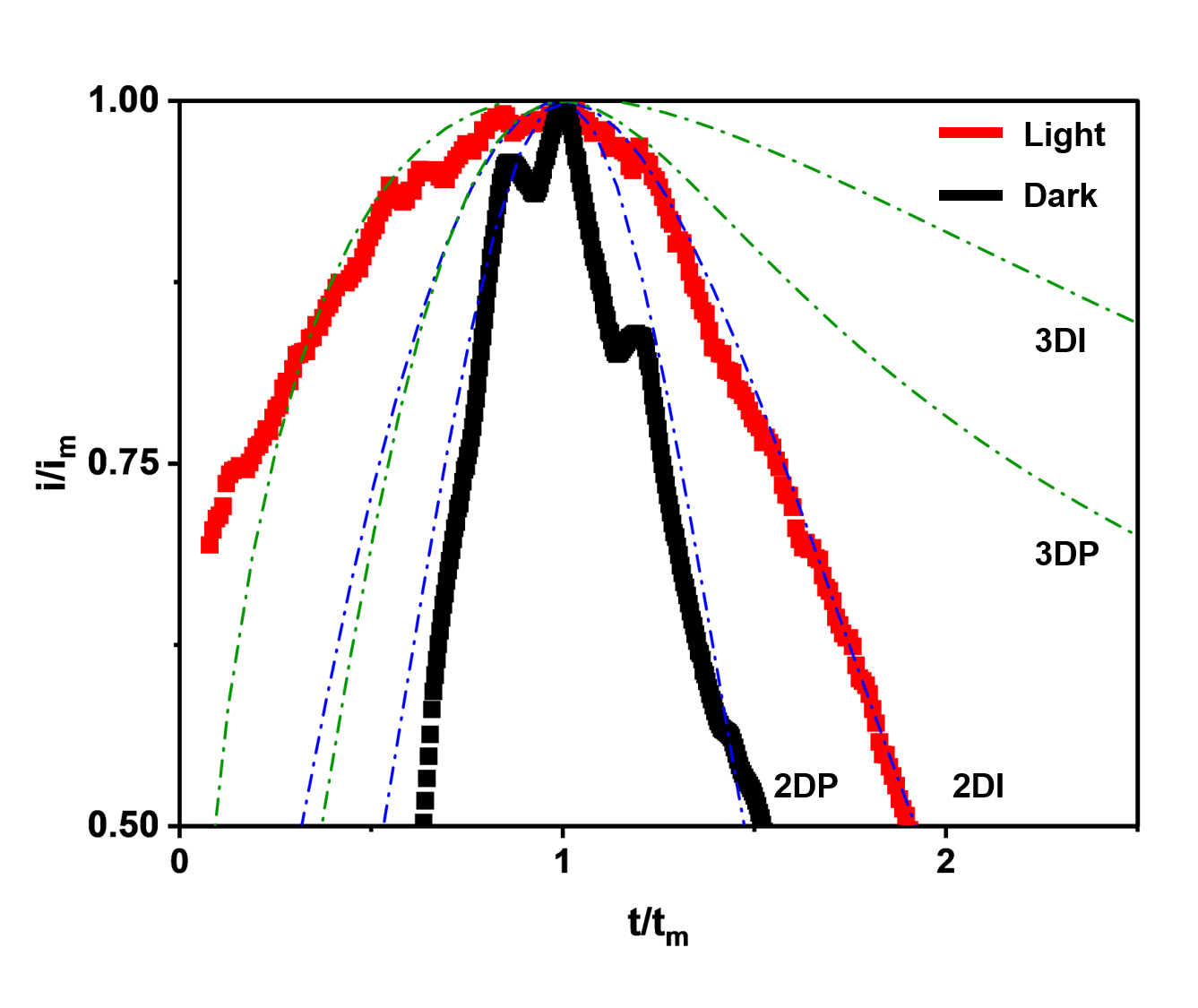


Figure S23. The dimensionless current–time transient for the Li_2_S deposited of the Au@N–TiO_2_ battery with and without the illumination.


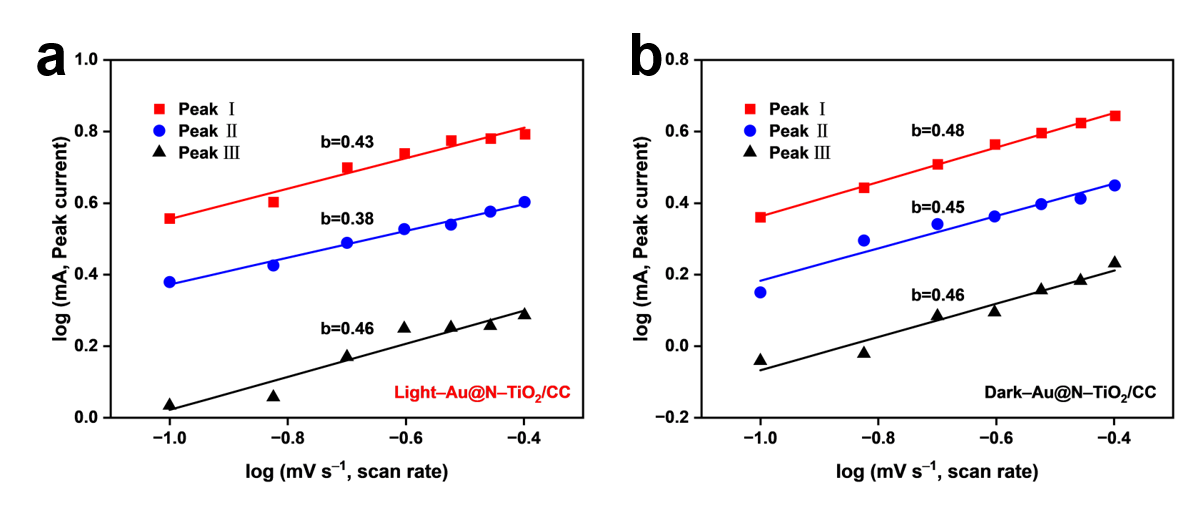


Figure S24. Linear relationships between peak current and scan rate a) with and b) without the light illumination.


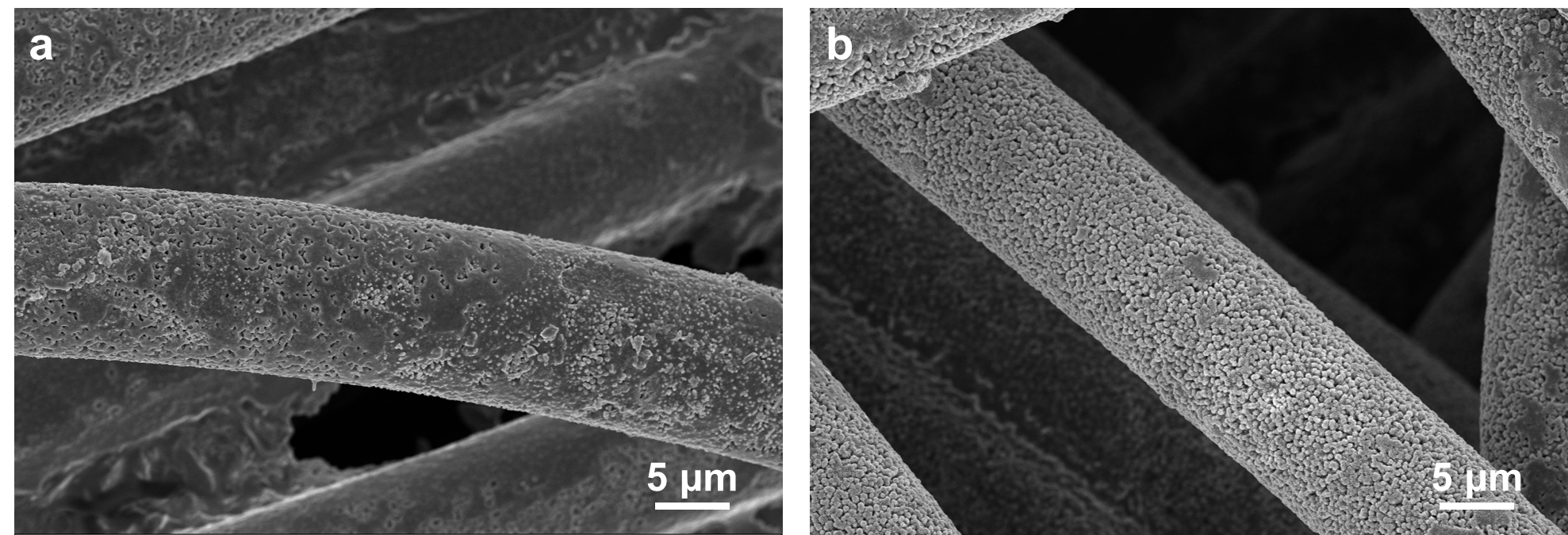


Figure S25. The SEM images of Au@N–TiO_2_/CC electrodes after 40 cycles (a) without the illumination and (b) with the illumination.


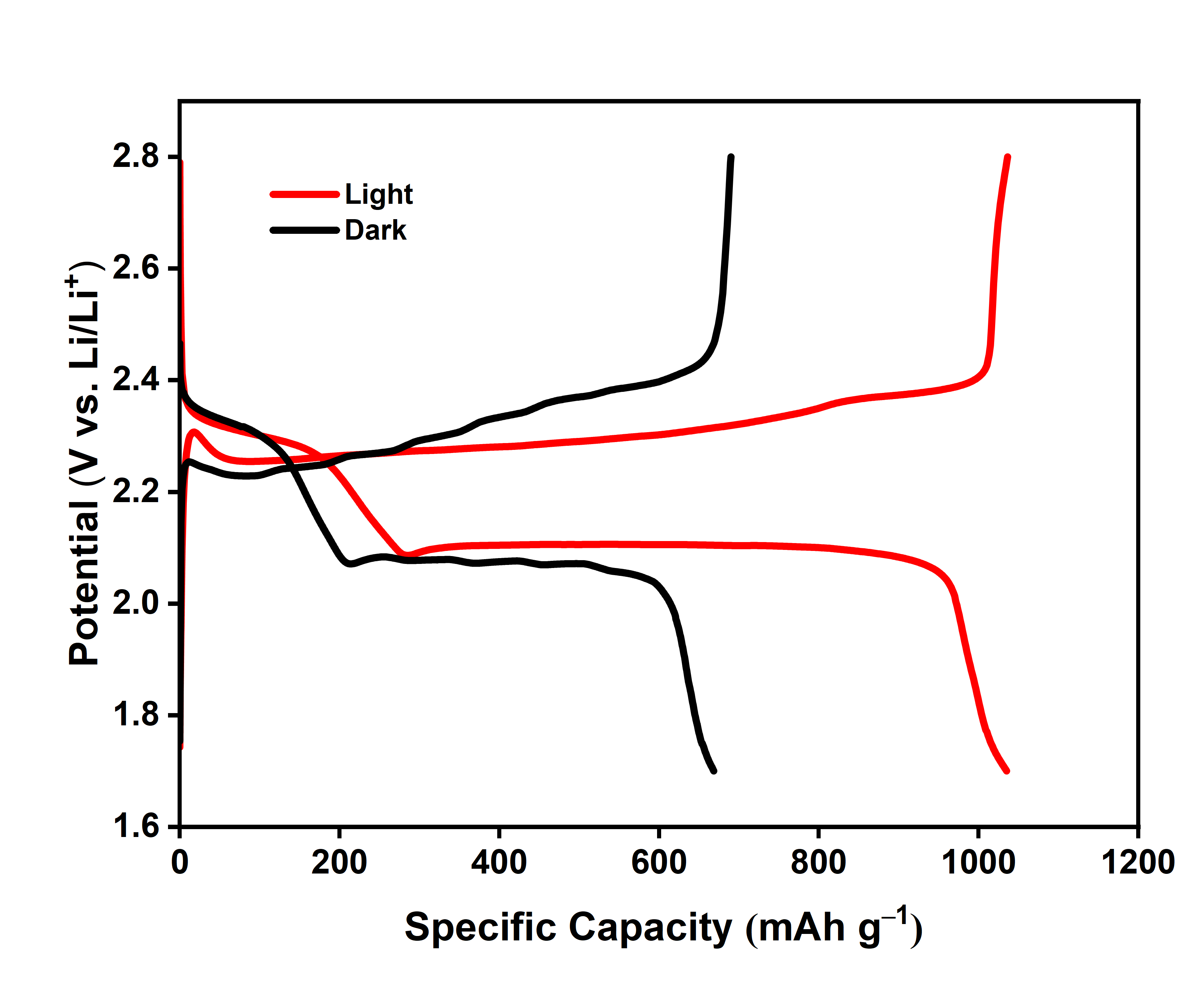


Figure S26. First GCD profiles of Au@N–TiO_2_/CC battery with and without the illumination with a high sulfur loading of 3.06 mg cm^–2^ at a rate of 0.1 C.


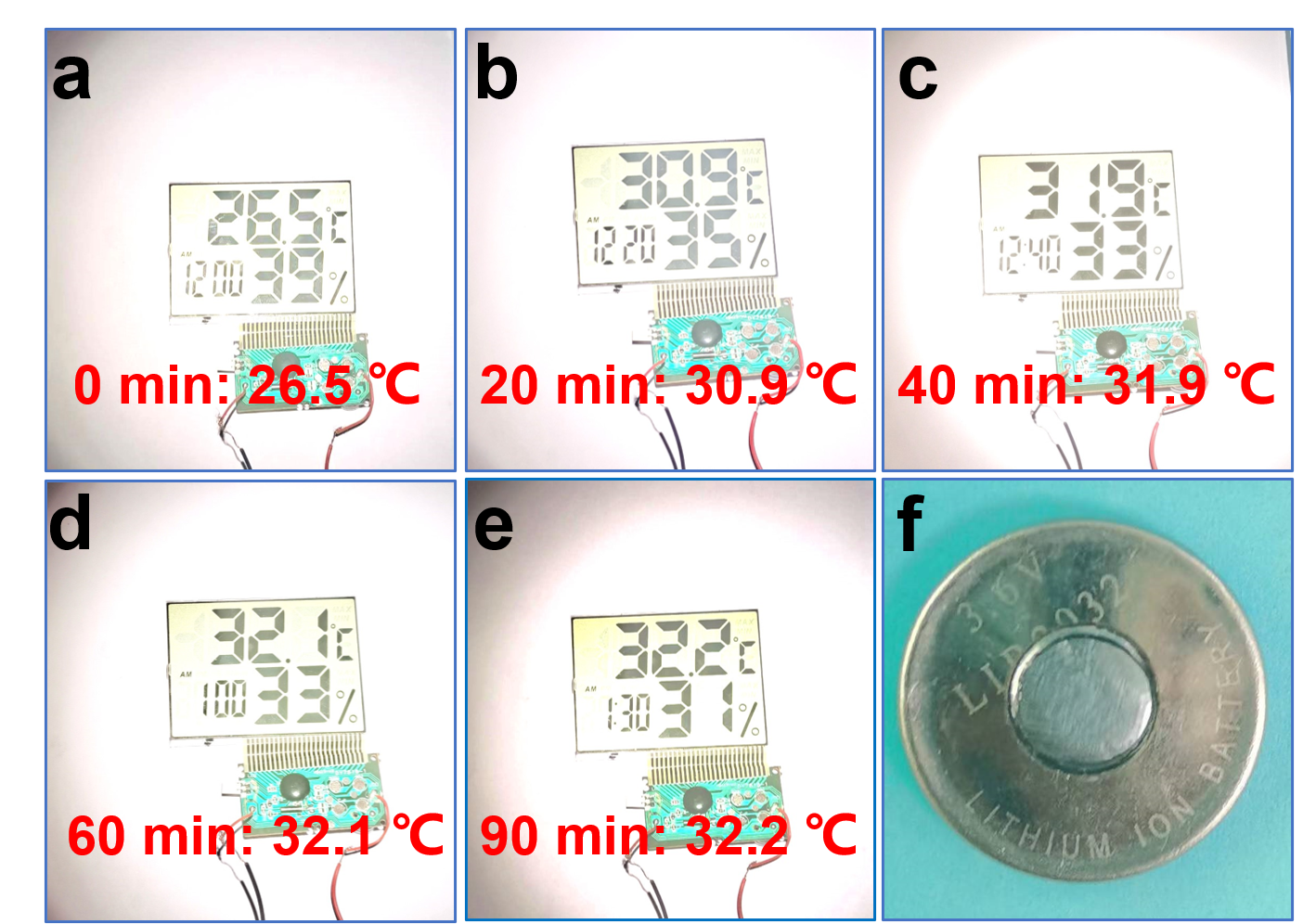


Figure S27. a–e) The plot of temperature change under light illumination. f) The PALSB used for testing the photothermal effect.


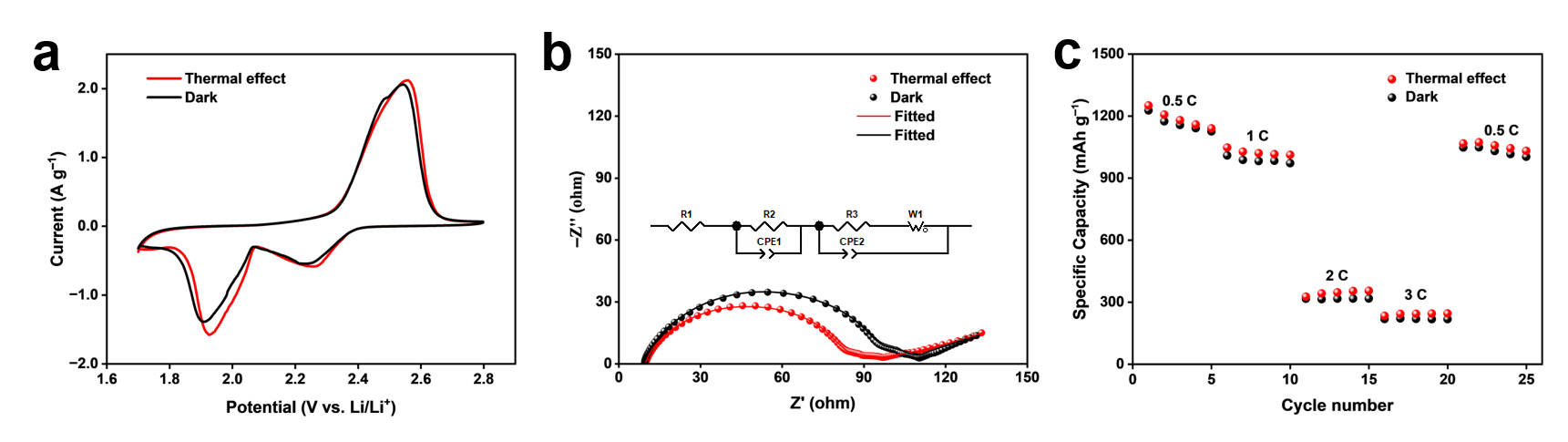


Figure S28. The photothermal effect on electrochemical performance of PALSB: a) CV curves at 0.1 mV s^−1^; b) EIS curves; c) Rate performance.


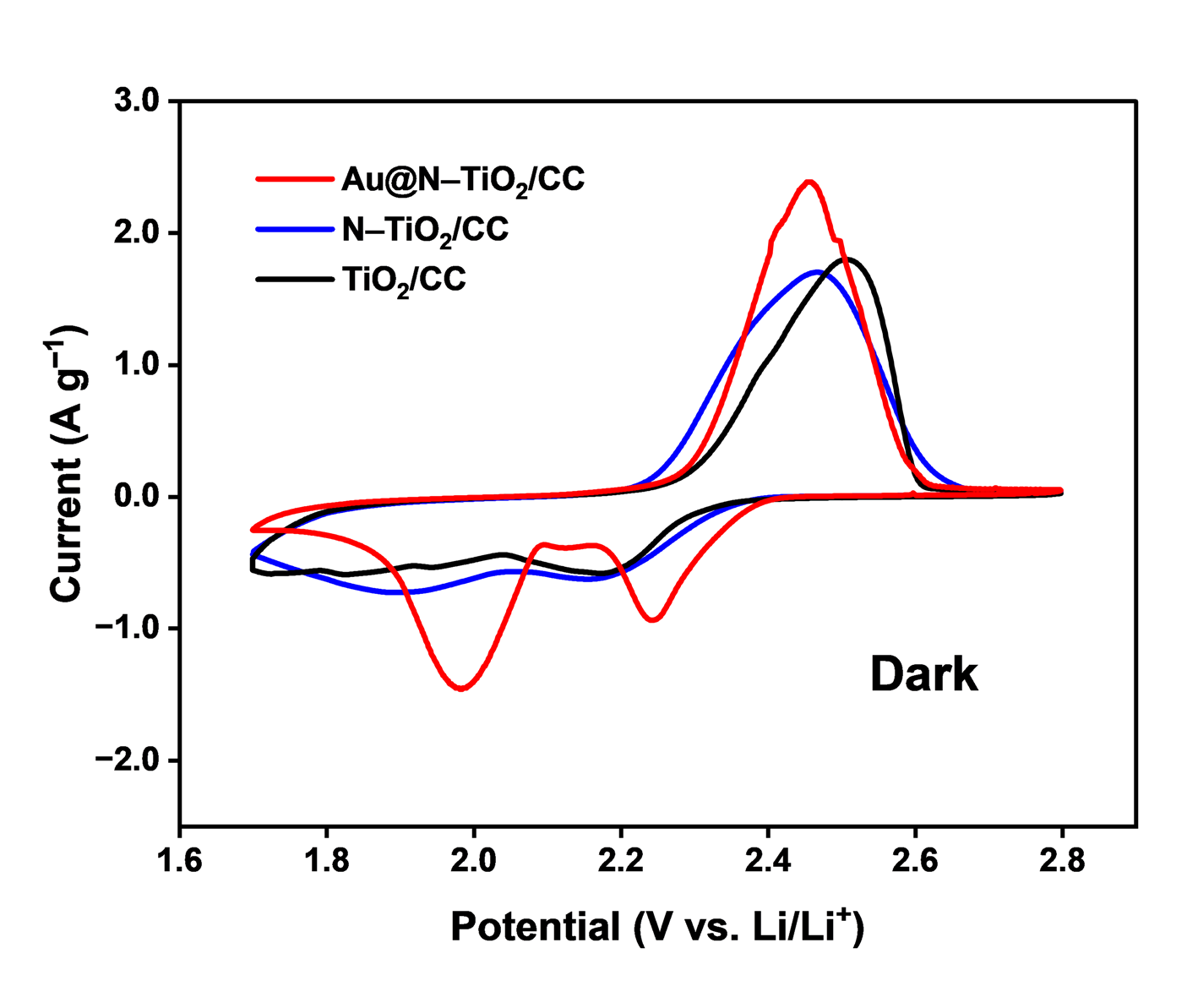


Figure S29. CV curves of LSB with various photoelectrodes without the illumination at

0.1 mV s^–1^


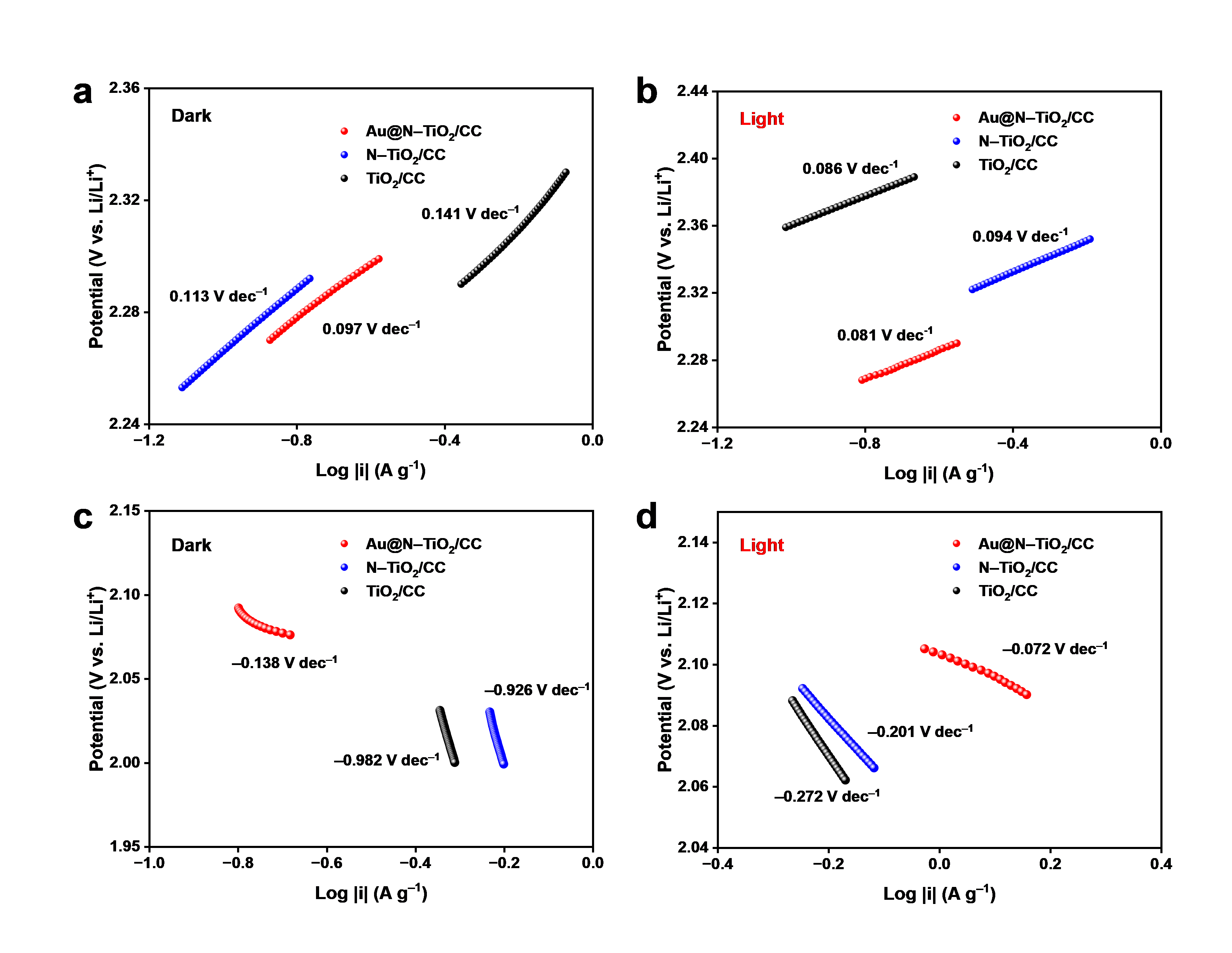


Figure S30. Tafel curves of various photoelectrodes a, c) without and b, d) without the illumination.


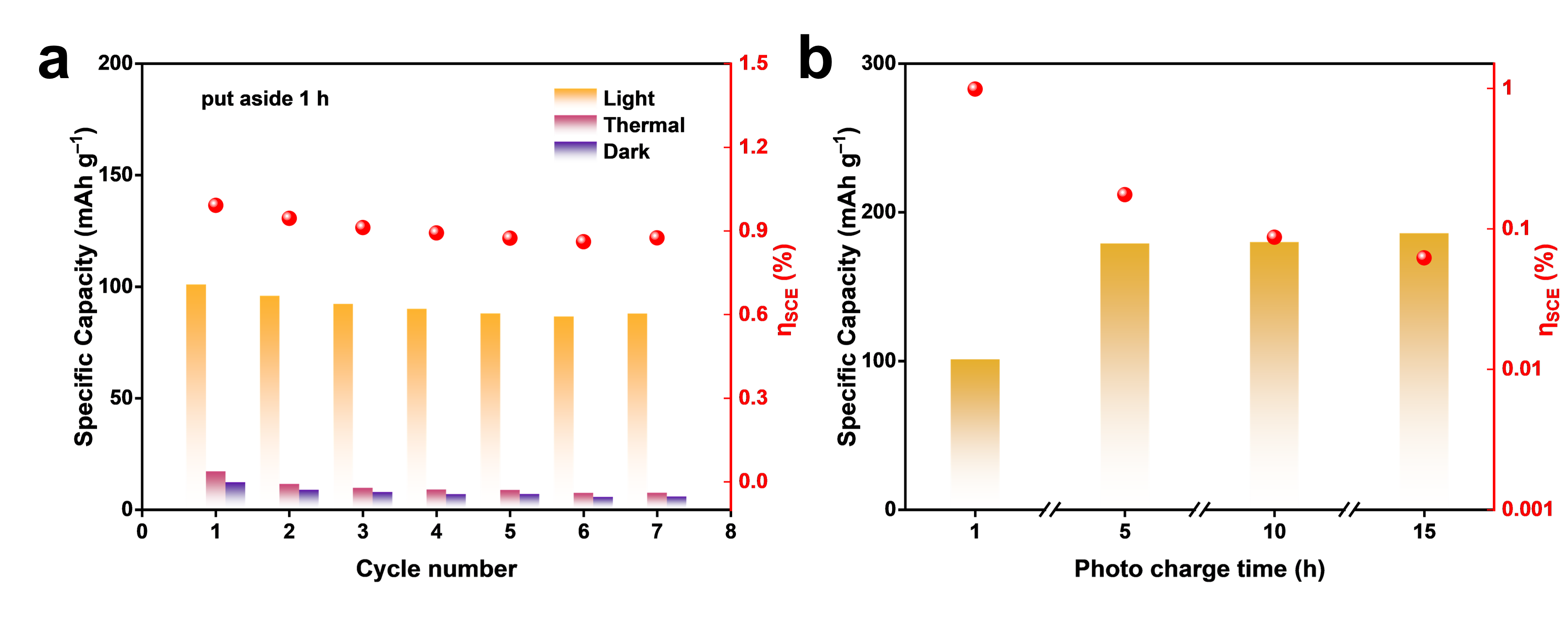


Figure S31. a) Cycling performance and solar energy conversion efficiency of PALSB after 1 h of photo–charging. b) the discharge capacity and solar energy conversion efficiency with different illumination time.


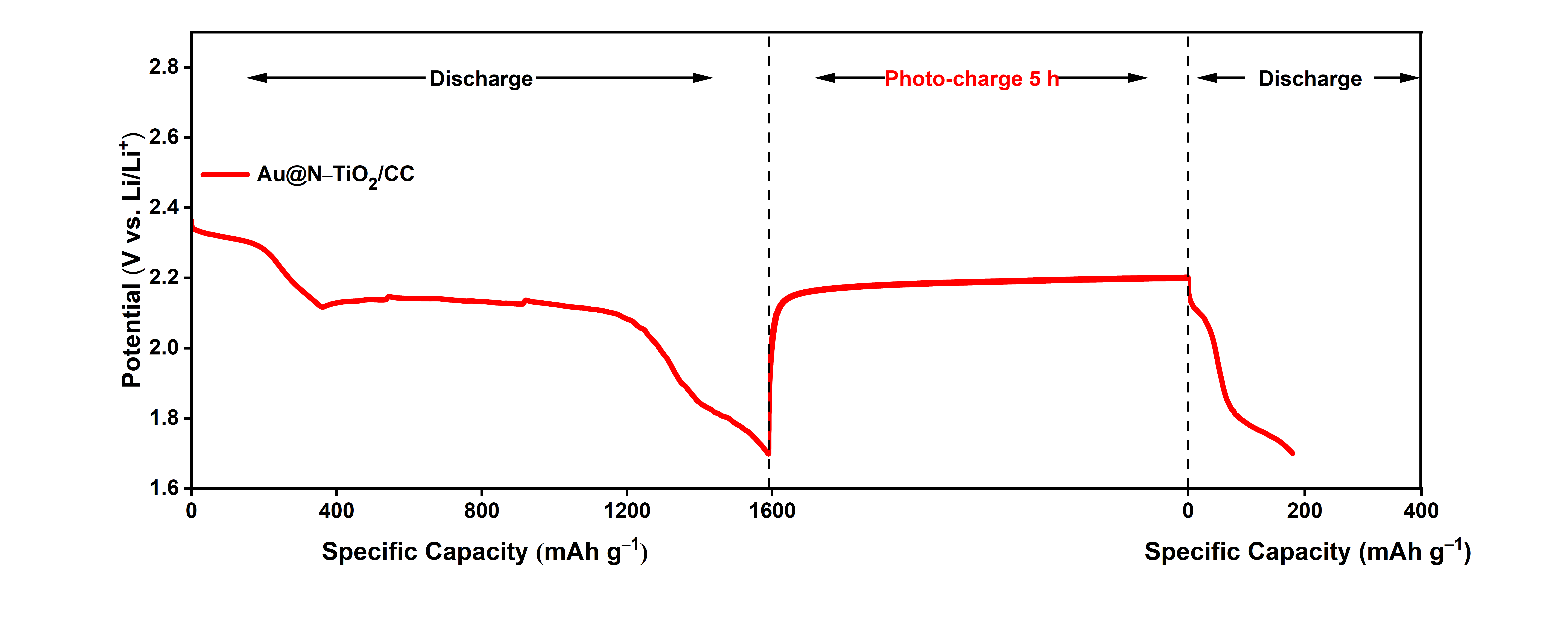


Figure S32. The initial discharge curve, and the discharge curve after 5 h of photo–charging of the PALSB.


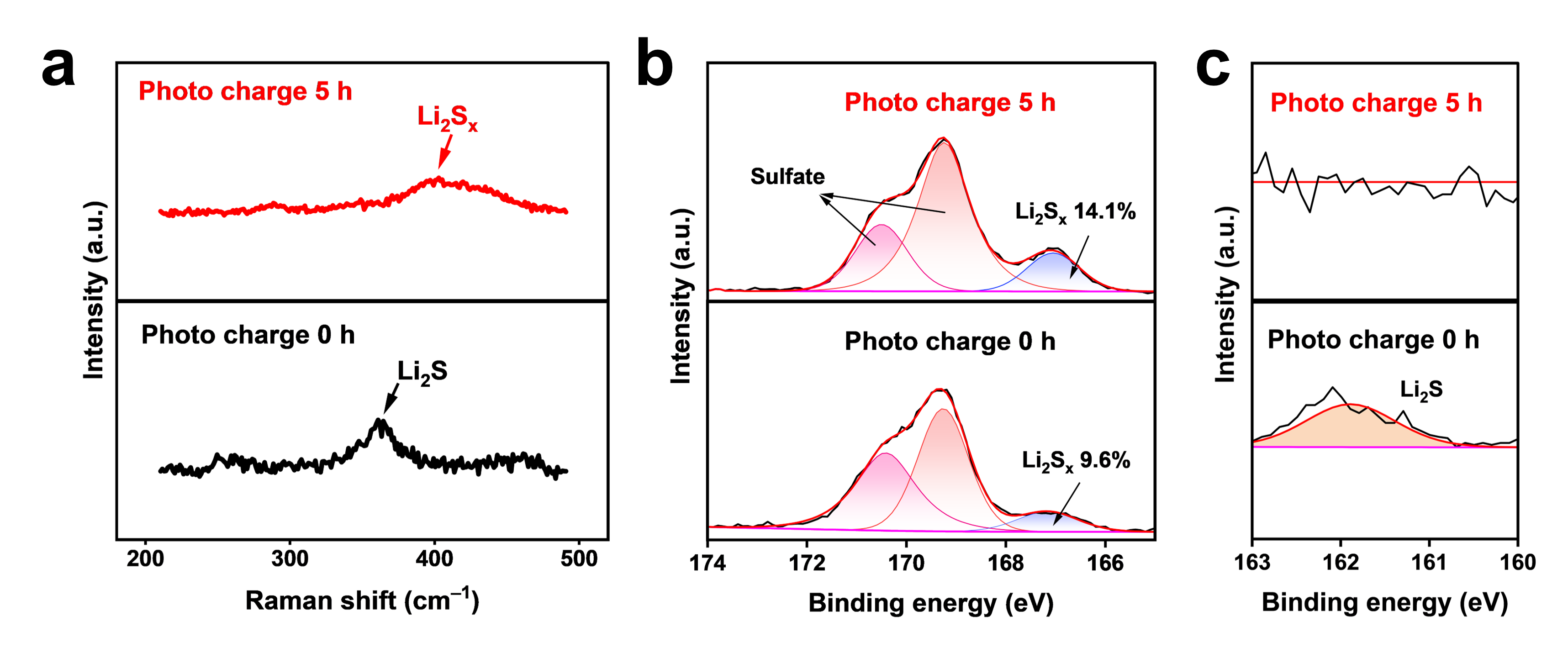


Figure S33. a) Raman and b, c) S 2p XPS spectra of Au@N–TiO_2_/CC assembled LSB at different photo charging stages.

Tabel S1. The fitted results from EIS analysis.

| Cathode | Test condition | R_1_ (ohm) | R_2_ (ohm) | R_3_ (ohm) |
| --- | --- | --- | --- | --- |
| Au@N–TiO_2_/CC | Dark | 9.9 | 88.1 | 7.1 |
|  | Thermal | 9.2 | 73.3 | 6.5 |
|  | Light | 7.2 | 13.1 | 3.4 |
| N–TiO_2_/CC | Light | 8.1 | 14.5 | 13.6 |
| TiO_2_/CC | Light | 10.1 | 28.0 | 13.9 |

Tabel S2. The comparisons of the comprehensive electrochemical performance of photo-assisted/charged LSBs with various cathodes.

| **Cathode** | **Current**  **density** | **Capacity**  **(mA h g^–1^)** | **Cycle**  **number** | **Fading**  **(per cycle)** | **Ref.** |
| --- | --- | --- | --- | --- | --- |
| CdS–TiO_2_/CC | 0.15 C | 1500 | 50 | 0.37% | [S2] |
| FTO/TiO_2_/S/N719 | 0.2 C | ~700 | 30 | 0.71% | [S3] |
| TiO_2_/dye//S/C | 0.3 C | 885 | 50 | 1.05% | [S4] |
| N719@TiO_2_//S@C | 2 C | 750 | 14 | no decay | [S5] |
| S–Li_7_P_2.9_Sb_0.1_S_10.75_O_0.25_–C | 0.1 C | 1482 | 10 | no decay | [S6] |
| Pt/CdS | 0.1 mA cm^–2^ | 792 | 10 | 0.75% | [S7] |
| **Au@N–TiO_2_/CC** | **3 C** | **855** | **50** | **0.16%** | **This work** |

Reference

[S1] Y. Chen, X. Tian, W. Zeng, X. Zhu, H. Hu, H. Duan, *J. Colloid Interface Sci.* 2015, *439*, 21.

[S2] Y.–H. Liu, J. Qu, W. Chang, C.–Y. Yang, H.–J. Liu, X.–Z. Zhai, Y. Kang, Y.–G. Guo, Z.–Z. Yu, *Energy Storage Mater.* 2022, *50*, 334.

[S3] J. Li, C. Ren, L. Zhang, W. Jiang, H. Liu, J. Su, M. Li, *J. Energy Chem.* 2022, *65*, 205.

[S4] Y. Qu, X. He, J. Hu, L. Duan, J. Wang, S. Liao, F. Lu, *J. Power Sources* 2023, *555*, 232374.

[S5] P. Chen, G.–R. Li, T.–T. Li, X.–P. Gao, *Adv. Sci.* 2019, *6*, 1900620.

[S6] T.–T. Li, Y.–B. Yang, B.–S. Zhao, Y. Wu, X.–W. Wu, P. Chen, X.–P. Gao, *Chem. Eng. J.* 2023, *455*, 140684.

[S7] N. Li, Y. Wang, D. Tang, H. Zhou, *Angew. Chem. Int. Ed.* 2015, *54*, 9271.
